# The structural basis for receptor recognition of human interleukin-18

**DOI:** 10.1038/ncomms6340

**Published:** 2014-12-15

**Authors:** Naotaka Tsutsumi, Takeshi Kimura, Kyohei Arita, Mariko Ariyoshi, Hidenori Ohnishi, Takahiro Yamamoto, Xiaobing Zuo, Katsumi Maenaka, Enoch Y. Park, Naomi Kondo, Masahiro Shirakawa, Hidehito Tochio, Zenichiro Kato

**Affiliations:** 1 Department of Molecular Engineering, Graduate School of Engineering, Kyoto University, Katsura, Nishikyo-ku, Kyoto 615-8510, Japan; 2 Department of Pediatrics, Graduate School of Medicine, Gifu University, Yanagido 1-1, Gifu 501-1194, Japan; 3 Graduate School of Nanobioscience, Yokohama City University, 1-7-29 Suehiro-cho, Tsurumi-ku, Yokohama Kanagawa 230-0045, Japan; 4 Institute for Integrated Cell-Material Sciences, Kyoto University, Kyoto 606-8501, Japan; 5 X-Ray Science Division, Argonne National Laboratory, 9700 South Cass Avenue, Argonne, Illinois 60439, USA; 6 Laboratory of Biomolecular Science and Center for Research and Education on Drug Discovery, Faculty of Pharmaceutical Sciences, Hokkaido University, , Kita-12, Nishi-6, Kita-ki, Sapporo 060-0812, Japan; 7 Research Institute of Green Science and Technology, Department of Bioscience, Graduate school of Science and Technology, Shizuoka University, 836 Ohya Suruga-ku, Shizuoka 422-8529, Japan; 8 Heisei College of Health Sciences, 180 Kurono, Gifu 501-1131, Japan; 9 Core Research of Evolution Science (CREST), Japan Sciences and Technology Agency, Tokyo 102-0076, Japan; 10 Department of Biophysics, Graduate School of Science, Kyoto University, Kitashirakawa-oiwake, Sakyo-ku, Kyoto 606-8502, Japan; 11 Biomedical Informatics, Medical Information Sciences Division, The United Graduate School of Drug Discovery and Medical Information Sciences, Gifu University, Gifu 501-1194, Japan

## Abstract

Interleukin (IL)-18 is a proinflammatory cytokine that belongs to the IL-1 family and plays an important role in inflammation. The uncontrolled release of this cytokine is associated with severe chronic inflammatory disease. IL-18 forms a signalling complex with the IL-18 receptor α (Rα) and β (Rβ) chains at the plasma membrane, which induces multiple inflammatory cytokines. Here, we present a crystal structure of human IL-18 bound to the two receptor extracellular domains. Generally, the receptors’ recognition mode for IL-18 is similar to IL-1β; however, certain notable differences were observed. The architecture of the IL-18 receptor second domain (D2) is unique among the other IL-1R family members, which presumably distinguishes them from the IL-1 receptors that exhibit a more promiscuous ligand recognition mode. The structures and associated biochemical and cellular data should aid in developing novel drugs to neutralize IL-18 activity.

## Abstract (web summary)

IL-18 is a member of the IL-1 family of proinflammatory cytokines. Tsutsumi *et al.* present a crystal structure of IL-18 bound to the extracellular domains of its heterodimeric receptor, providing insight into how its unusual specificity may be targeted pharmacologically.

## Graphical abstract

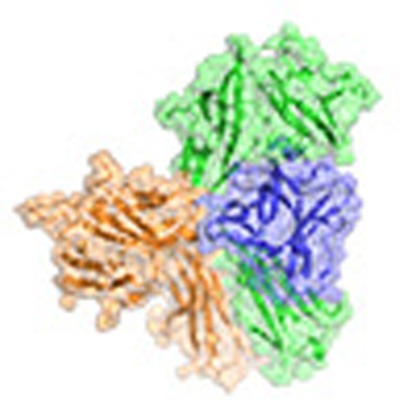

Interleukin (IL)-18 belongs to the IL-1 superfamily and was first discovered as an interferon gamma (IFN-γ)-inducing factor in sera from mice with hepatitis stimulated with *Propionibacterium* acnes and lipopolysaccharide^1^. This proinflammatory cytokine is secreted by various types of cells and strongly augments IFN-γ production in type-1 helper T (Th1) cells and natural killer (NK) cells following activation of NK-cell cytotoxicity; thus, it plays a critical role in inflammation and the host defense against microbes. In addition to IL-1β^2^,^3^, IL-18 is synthesized as a biologically inactive precursor (proIL-18) on activation of a certain class of receptors, such as Toll-like receptors and proinflammatory cytokine receptors, and then stored in the cytosol. Once it matures via caspase-1 (ref. 4), which is regulated by a large protein complex referred to as the inflammasome^5^, IL-18 is extracellularly secreted and binds IL-18 receptor α (Rα) as well as IL-18 receptor β (Rβ) at the immunocyte plasma membrane in a stepwise manner. IL-18/IL-18Rα/IL-18Rβ ternary complex formation juxtaposes the intracellular Toll-Interleukin-1 receptor domains of IL-18Rα and IL-18Rβ, to which the adaptor molecule myeloid differentiation factor 88 (MyD88) is recruited presumably with the aid of TRAM^6^. MyD88 further interacts with IL-1 receptor associating kinase (IRAK) 4 and IRAK1/2 to form the large molecular assembly referred to as Myddosome, which subsequently activates IKK via TRAF6. Finally, the signal activates the NF-κB and mitogen-activated protein kinase pathways^7^, which upregulate the expression of various inflammatory cytokines.

Of the IL-1 family cytokines, IL-18 and IL-1β have garnered much attention because they are causal cytokines that lead to severe chronic inflammatory syndrome. IL-1β is associated with immunological disorders, such as autoinflammatory syndromes^8^,^9^. The central pathogenic feature of autoinflammatory syndromes is excess production of mature IL-1β derived from abnormal inflammasome activation due to certain gene mutations. IL-1β-related autoinflammatory diseases are treated through neutralizing IL-1β by anti-IL-1β (canakinumab and gevokizumab), engineered soluble receptors (rilonacept) or the receptor antagonist IL-1Ra (anakinra), which is remarkably effective; thus, these treatments are currently in clinical use^10^. Similar to IL-1β, IL-18 overproduction likely leads to severe autoimmune, autoinflammatory, allergic, neurological and metabolic disease, which might be associated with IL-18 or IL-18 receptor genetic polymorphisms^11^,^12^,^13^,^14^. Two recent papers have revealed that constitutive activation of the inflammasome caused by single point mutations in NLRC4 is associated with a novel autoinflammatory disorder, and the patient with NLRC4-mediated macrophage activation syndrome showed ultra-high circulation levels of IL-18 even after IL-1 blockade^15^,^16^. Consistent with these observations, therapeutic approaches that block IL-18 activity have been effective in inflammatory disease models^17^,^18^. Therefore, developing drugs that impede binding between IL-18 and the receptors is clinically important. Generally, the atomic structures of targeted proteins and their complexes play vital roles in drug design. Thus far, despite the reported structures for free IL-18 and its related complexes^19^,^20^,^21^,^22^, a structure for the genuine complex between IL-18 and its receptors has not yet been determined.

Previously, we reported a solution structure for IL-18 and identified the functional residues for which mutation markedly decreased its binding affinity for IL-18Rα^19^. The results suggest that the binary complex between IL-18 and IL-18Rα exhibits an essentially identical binding mode to the complex between IL-1β and its receptors (IL-1RI or IL-1RII). However, the binding mode for IL-18Rβ, which is the IL-18 co-receptor, to IL-18/IL-18Rα remained ambiguous. Recent structural studies on the ternary complex between IL-1β and its receptors’ ectodomains^23^,^24^ demonstrate that IL-1RAcP, which is the commonly used co-receptor for IL-1α, IL-1β, IL-33 and IL-36s, adopted a ‘left’ binding mode. In this mode, IL-1RAcP binds the IL-1β/IL-1RI or IL-1β/IL-1RII binary complexes from the left side as seen from the concave IL-1β recognition surface of IL-1RI or IL-1RII. Furthermore, the other IL-1 superfamily molecule, IL-33/ST2/IL-1RAcP, was also suggested to adopt the ‘left’ binding mode based on the model structure from the small angle X-ray scattering (SAXS) profiles^25^. Thus, left binding seems common in complexes that employ IL-1RAcP. In contrast to other IL-1 family cytokines, IL-18 is unique due to its pair of specialized receptors (IL-18Rα and IL-18Rβ); hence, the recognition details are not sufficiently understood based only on homology to the IL-1β and IL-33 system.

Here, we performed X-ray crystallography using human IL-18 and its complexes with the receptors’ extracellular domains. The structures demonstrate that the co-receptor (IL-18Rβ) binding mode is generally identical to IL-1β; however, substantial differences were observed in the subdomain orientations and interaction details throughout the complex. Intriguingly, the second domain (D2) of the two IL-18 receptors lacked one β-strand, d2, which is conserved among other IL-1-related receptors, and was previously shown to contribute to the inter-receptor interaction. In addition, N-linked glycans played a role in bridging the two receptors, which was observed in the signalling IL-1β receptor complex but was absent in its decoy complex. We further show that other IL-18Rα N-linked glycans proximal to IL-18 in the complexes contributed to the binding affinity. With the associated biochemical and cell biological data, the structures comprehensively clarify the IL-18 receptor recognition mode, which will facilitate rational drug development to neutralize IL-18 activity, the uncontrolled release of which has been shown to cause severe chronic inflammatory diseases.

## Results

### Structural comparison between the IL-18 and IL-1β complexes

**Figure 1:**
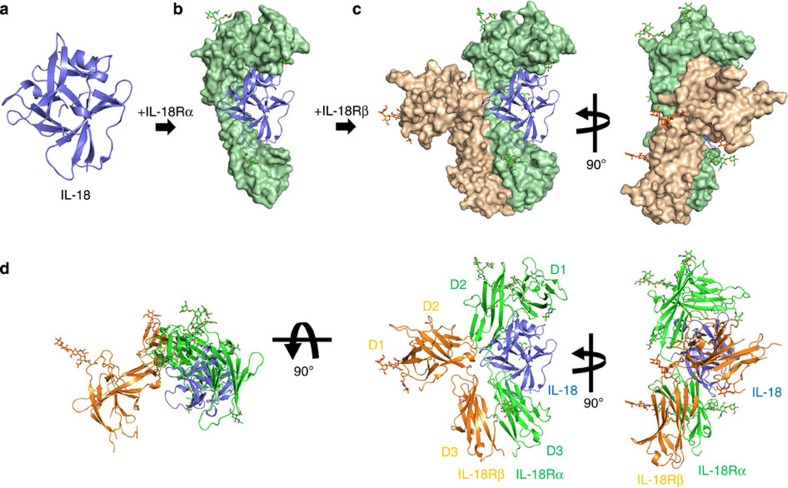
Overall structures for IL-18 and its extracellular complexes. (**a**–**c**) Schematic flow diagram of the stepwise complex formation for IL-18/IL-18Rα/IL-18Rβ. Crystal structures of (**a**) IL-18, (**b**) IL-18/IL-18Rα and (**c**) IL-18/IL-18Rα/IL-18Rβ are shown as a ribbon (IL-18, blue) or surface (IL-18Rα, palegreen; IL-18Rβ, wheat) representation, respectively. (**d**) Ribbon diagrams of the ternary complex structure for IL-18 (blue), IL-18Rα (green) and IL-18Rβ (orange) from three perspectives.

We determined the crystal structures of IL-18 (Fig. 1a), the IL-18/IL-18Rα binary complex (Fig. 1b) and the IL-18/IL-18Rα/IL-18Rβ signalling ternary complex (Fig. 1c,d) at the resolutions 2.33, 3.10 and 3.10 Å, respectively. The crystallographic statistics are provided in Table 1.

**Figure 2:**
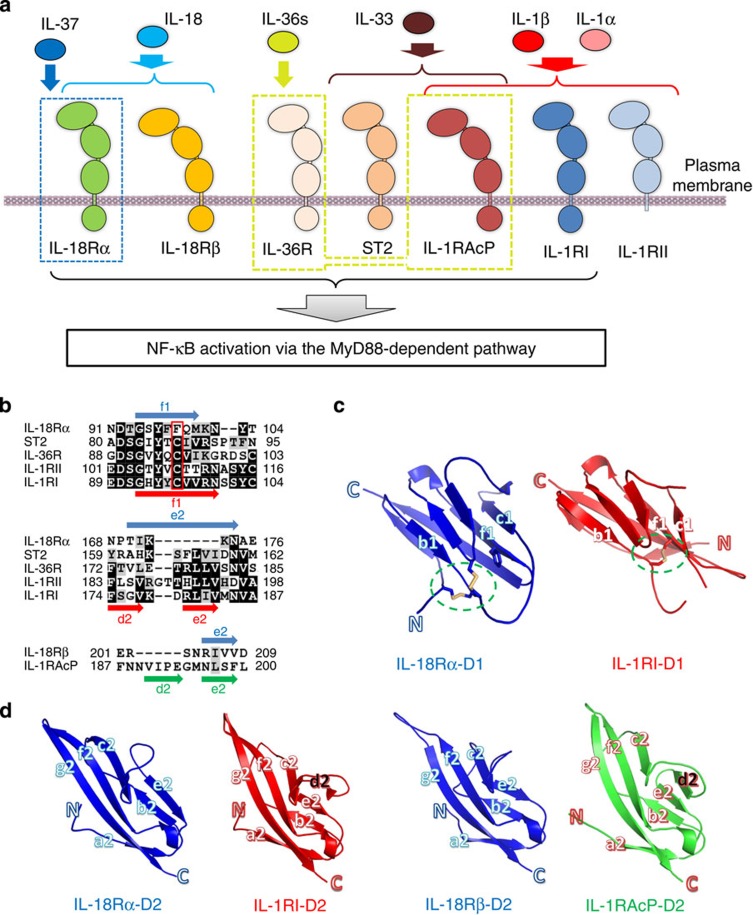
Structural features of IL-18 and its receptors. (**a**) Promiscuous interactions between the IL-1 family agonists and IL-37 with the receptor molecules. Except IL-18, all agonists employ IL-1RAcP as a co-receptor, while IL-18 uses IL-18Rβ. (**b**) Certain regions of the receptor multiple sequence alignments manifest the unique qualities of IL-1R family IL-18Rs. The full set of sequence alignments is in Supplementary Fig. 1. (**c**) Structural comparison of D1 and (**d**) D2 of the receptors from the signalling complex. The green circles in (**c**) show the disulfide bond positions.

IL-18Rα curls around IL-18, and IL-18Rβ contacts the lateral portion of the IL-18/IL-18Rα binary complex in a similar manner as the IL-1β/IL-1RI(RII)/IL-1RAcP complex^23^,^24^. The IL-18 structure essentially does not change on complex formation, maintaining the β-trefoil fold that comprises 12 β-strands (β1-β12) and 2 α-helices (α1-α2) (Supplementary Fig. 1a), as previously reported^19^. The IL-18Rα ectodomain folds into three immunoglobulin (Ig)-like domains, which are referred to as D1, D2 and D3, in the same manner as the IL-1 receptors^23^,^24^,^26^. Each domain comprises a two-layer sandwich of six to nine β-strands and contains at least one intra-domain disulfide bond (Supplementary Fig. 1b). Within IL-18Rα, D1 extensively contacts D2, whereas D3 is distant and is connected by the long D2-D3 linker (Fig. 1d middle), which implies that D1 and D2 behave as a single module, similar to IL-1-related primary receptors. In the Ig superfamily, including the IL-1 receptor family (Fig. 2a), the core cysteine residues on the b and f strands are highly conserved (Fig. 2b). However, for IL-18Rα-D1, the f strand cysteine is replaced with phenylalanine (Fig. 2b), which yields two unexpected surface disulfide bonds (Fig. 2c). In addition, the D2 domain lacks one β-strand (d2 in Fig. 2b,d) that is structurally conserved among most IL-1 receptor family members, IL-1RI, IL-1RII and ST2 as well as IL-1RAcP.

**Figure 3:**
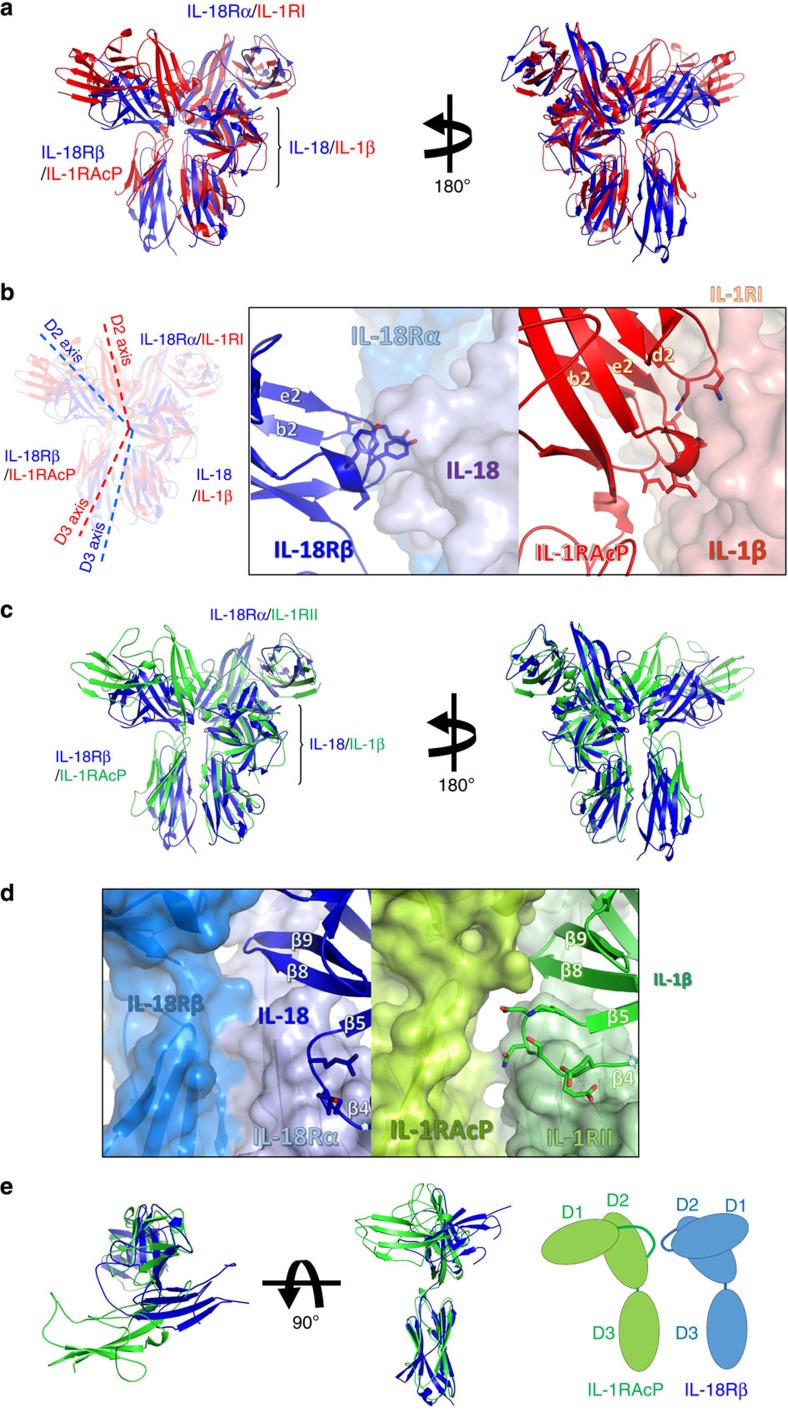
Structural comparison of the IL-1 family cytokine ternary complexes. (**a**) Structural alignment of IL-18/IL-18Rα/IL-18Rβ (blue) and IL-1β/IL-1RI/IL-1RAcP (4DEP, red) using the binary complex region. The backbone Cα RMSD between IL-18/IL-18Rα and IL-1β/IL-1RI is 4.35–4.36 Å. (**b**) The orientation of D2 and D3 from IL-18Rβ and IL-1RAcP in the complexes. The dotted lines show the approximate orientation of the longitudinal D2 and D3 axes. A close-up of the binary complex interface, which recognizes the co-receptor protein D2 domains, is also shown. (**c**) A comparison of IL-18/IL-18Rα/IL-18Rβ (blue) and IL-1β/IL-1RII/IL-1RAcP (3O4O, green). The backbone Cα RMSD was 4.39 Å surrounding the binary complex portion. (**d**) A close-up of the β4-β5 loops of the ligands in IL-18/IL-18Rα/IL-18Rβ (left) and IL-1β/IL-1RII/IL-1RAcP (right). (**e**) Superimposition of IL-18Rβ and IL-1RAcP (from 3O4O). The IL-18Rβ-D1 orientation relative to D2 is the opposite of IL-1RAcP.

On ternary complex formation, the IL-18/IL-18Rα binary complex structure essentially does not change; the root mean square deviations (RMSD) for the backbone Cα atoms are 0.41–0.90 Å compared with the asymmetric unit (ASU) molecules. IL-18Rβ is also composed of three Ig-like domains, similar to IL-18Rα except for the aforementioned disulfide bonds (Supplementary Fig. 1c); however, the spatial arrangement of these domains is markedly distinct from IL-18Rα in the ternary complex (Fig. 1d). D2 and D3 of IL-18Rβ are close to each other and directly associated with IL-18/IL-18Rα, while D1 does not contribute to ternary complex formation. In fact, a part of the IL-18Rβ-D1 electron density is ambiguous presumably because the region was loosely packed in the crystal (Supplementary Fig. 2). The D1 isolation of the co-receptor was also observed previously in the IL-1β/IL-1RI(RII)/IL-1RAcP complex, where IL-1RAcP-D1 is outstretched and does not participate in the molecular interface. Despite of the similarity in the binding mode, relative orientation of IL-18Rβ-D2+D3 to IL-18/IL-18Rα is different from that of IL-1RAcP-D2+D3 to IL-1β/IL-1RI(RII) (Fig. 3a–c). This difference in the orientations is attributable to the interaction manner at the interface between IL-18Rβ-D2 and IL-18/IL-18Rα (Fig. 3b,d). Owing to the aforementioned absence of the d2 strands in D2s (Fig. 2b,d), IL-18Rα-D2 and IL-18Rβ-D2 supply only two strands (b2 and e2) and loops at the interface, respectively, while for IL-1RI(RII) and IL-1RAcP-D2, three strands (b2, e2 and d2) and loops interact (Fig. 1d and Fig. 3a–c). Furthermore, IL-18Rβ-D2 supplies not only electrostatic side chains but also aromatic rings that interact with IL-18, whereas IL-1RAcP-D2 utilizes electrostatic and aliphatic side chains for ligand binding. The β4-β5 loop of IL-18 and IL-18Rβ-D3 do not interact, which may also contribute to the orientation difference (Fig. 3d). A more marked dissimilarity is observed for the IL-18Rβ-D1 orientation relative to -D2, which differs from IL-1RAcP in the complex (Fig. 3e). As a result, despite the same ‘left’ binding mode, the spatial arrangements of the subdomains in the IL-18/IL-18Rα/IL-18Rβ ternary complex differ somewhat from IL-1β/IL-1RI/IL-1RAcP with Cα atom RMSD values of 6.64–6.73 Å.

### The binding interface between IL-18 and IL-18Rα

**Figure 4:**
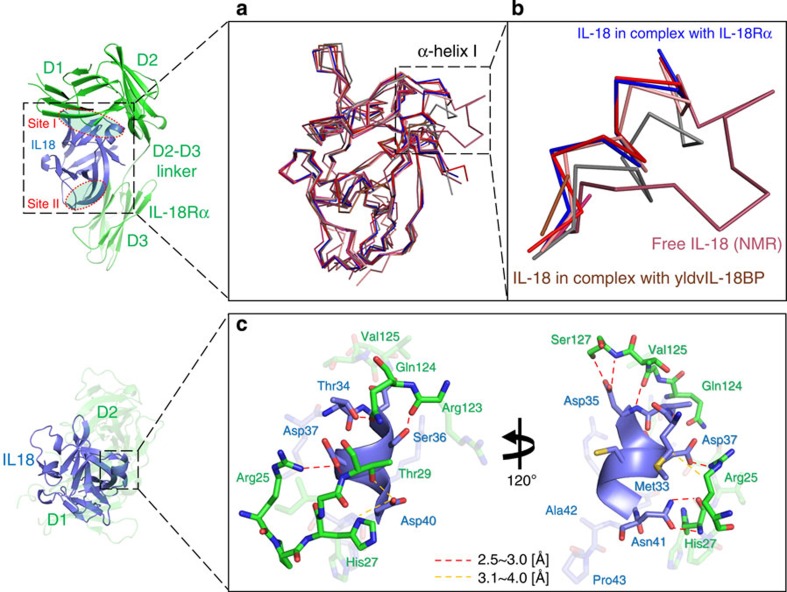
IL-18 structural perturbations on binding IL-18Rα or other proteins. (**a**) Superimposition of the structures of IL-18 in free and various complex forms: crystal structure of IL-18 with CHPAS (red); the solution structure (1J0S, raspberry); the crystal structure of IL-18 in complex with Ectromelia virus IL-18BP (3F62, pink); the crystal structure of IL-18 complexed with murine reference antibody 125-2H Fab (2VXT, salmon); the crystal structure for IL-18 in complex with YLDV 14L IL-18BP (4EEE, brown); the crystal structure for IL-18 in complex with a DVD-Ig molecule (4HJJ, gray); the crystal structure of IL-18 in complex with IL-18Rα (blue). (**b**) The zoom window of the dashed line box shown in 2**a**. (**c**) The stabilized structure and IL-18 α-helix I interactions. The structure of IL-18 α-helix I stabilized by interactions with IL-18Rα.

IL-18Rα recognizes IL-18 through a large interaction surface area at ~1,850 Å^2^. Two IL-18 sites, Sites I and II, contact IL-18Rα; Site I is located on a side of the core barrel of the β-trefoil structure, and Site II is at the top of the β-barrel (Fig. 4a).

Our previous NMR study showed that the residues 34 to 42 are flexible despite partial α-helix formation in this region (residues 38–41)^19^. In addition, two isomeric forms that originate from a *cis* or *trans* peptide bond between Ala42^IL-18^-Pro43^IL-18^ in the loop are equally populated in the solution structure. The flexible nature of the segment produces a variety of architectures, as demonstrated in several crystallographic reports, including a loop with a *trans* isomer^22^, an α-helix with a *cis* isomer^21^ or, primarily, unobservable flexible loop with a *trans* isomer^20^,^22^ (Fig. 4a–c). In both the binary and ternary complexes, the segment between residues 35 and 40 adopts an α-helix structure with a *cis* Ala42^IL-18^-Pro43^IL-18^ bond, which is stabilized by hydrogen bonds and electrostatic interactions with IL-18Rα (Fig. 4c).

**Figure 5:**
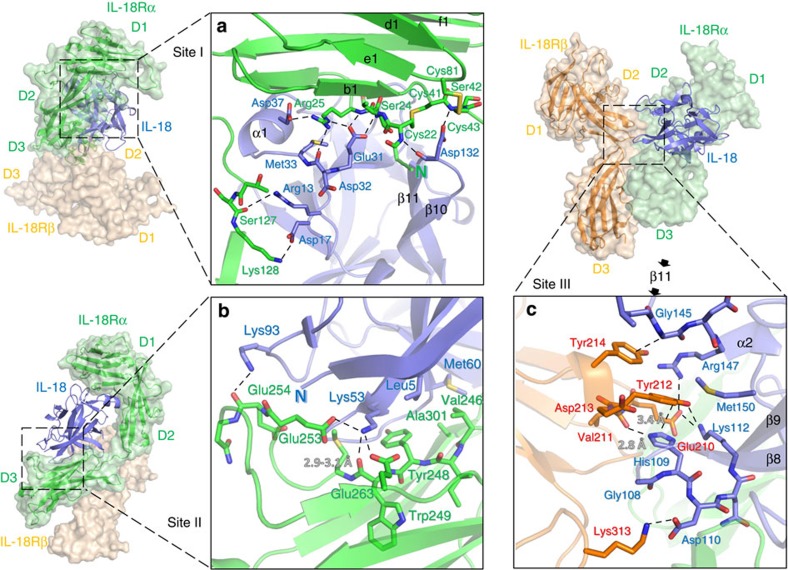
The key residues involved in forming the ternary signalling complex. (**a**) The interactions in the IL-18 receptor binding Site I. In addition to the area surrounding the stable α-helix I, Asp132^IL-18^ at the tip of the β10-β11 hairpin exhibits ionic interactions with the structure, which are formed by the two unique disulfide bonds in IL-18Rα. (**b**) Interactions surrounding the IL-18 Site II. The key residue of Lys53^IL-18^ is recognized by the IL-18Rα acidic surface through multiple hydrogen bonds. (**c**) The interface between Site III of IL-18 and IL-18Rβ. In addition to His109^IL-18^/Tyr212^Rβ^ stacking, multiple hydrogen bonds were observed: the His109^IL-18^ Nε-H to the Val211^Rβ^ backbone carbonyl oxygen as well as the Lys112^IL-18^ Nζ-H to the Glu210^Rβ^ side chain and Tyr212^Rβ^ Oη.

Surrounding Site I, α-helix I mediated the interaction (Fig. 4c and Fig. 5a), wherein the Arg25^Rα^ side chain is buried within the β3-α1 acidic groove of IL-18. Two disulfide bonds, Cys22^Rα^-Cys41^Rα^ and Cys43^Rα^-Cys81^Rα^, bridge the b1-c1 loop to the N-terminal loop and d1-e1 turn, respectively (Figs 2c and 5a), which likely reinforce the proximal loop structure, to which the long β10-β11 hairpin of IL-18 is fitted. This unique feature implies that the loop structure may be loosened under certain reductive conditions, which affects the affinity for the ligand. The Ser42^Rα^ amide proton supplies a hydrogen bond for the Asp132^IL-18^ side chain, which also exhibited a backbone–backbone hydrogen bond with Cys22^Rα^. Around Site II (Fig. 5b), the IL-18Rα acidic surface, which is composed of the Glu253^Rα^ and Glu263^Rα^ carboxylates as well as Trp249^Rα^ backbone oxygen, captures the Lys53^IL-18^ ε-amino group.

### IL-18Rβ recognition by IL-18/IL-18Rα

The IL-18/IL-18Rα/IL-18Rβ signalling ternary complex is formed by IL-18Rβ binding with the lateral portion of the binary complex (Fig. 1). The IL-18Rβ-D2 convex surface with the key Tyr212^Rβ^ aromatic residue fits into the concave surface jointly formed by IL-18 and IL-18Rα-D2 (Fig. 5c and Supplementary Fig. 3a) with a 613 Å^2^ buried surface area, which is shallower than that in the IL-1β complexes. The concave surface area is divided into Site III on IL-18 (354 Å^2^, Fig. 5c) and part of IL-18Rα-D2 (259 Å^2^, Supplementary Fig. 3a). Note that IL-18 Site III is revised in this work^19^. Site III of IL-18 comprises the prominent β8-β9 hairpin and β11-α2 loop (Fig. 5c), where the aromatic ring of His109^IL-18^ forms π-π stacking with Tyr212^Rβ^ at a 3.4 Å distance, which is surrounded and stabilized by multiple hydrogen bonds. IL-18Rα-D2 is composed of an antiparallel β-sheet formed by b2 and e2 (Supplementary Fig. 3a) but lacks the conserved d2 strand compared with the corresponding β-sheets of IL-1RI and ST2. Although the structure of IL-36R has not been determined yet, the multiple sequence alignment suggests that the b2/e2/d2 sheet is conserved among the primary receptors (Fig. 1b and Supplementary Fig. 1b). In addition to these interactions, IL-18Rβ-D3 extensively contacts IL-18Rα-D3 with the buried area ~550 Å^2^, to which both electrostatic (Supplementary Fig. 3b) and hydrophobic interactions (Supplementary Fig. 3c) contribute. These inter-receptor interfaces in the IL-1β complex are designated as Site IV^23^, in which the IL-1β β4-β5 loop interacts with IL-1R1-D3 and IL-1RAcP-D3 to at least partly establish the ligand’s agonism/antagonism. The same would be true for the IL-36 system based on the crystal structure of IL-36 (ref. 27). In contrast, the corresponding IL-18 loop does not contact IL-18Rβ-D3 and only interacts with IL-18Rα-D3 (Fig. 3d).

### N-linked IL-18Rα glycans and its interactions

**Figure 6:**
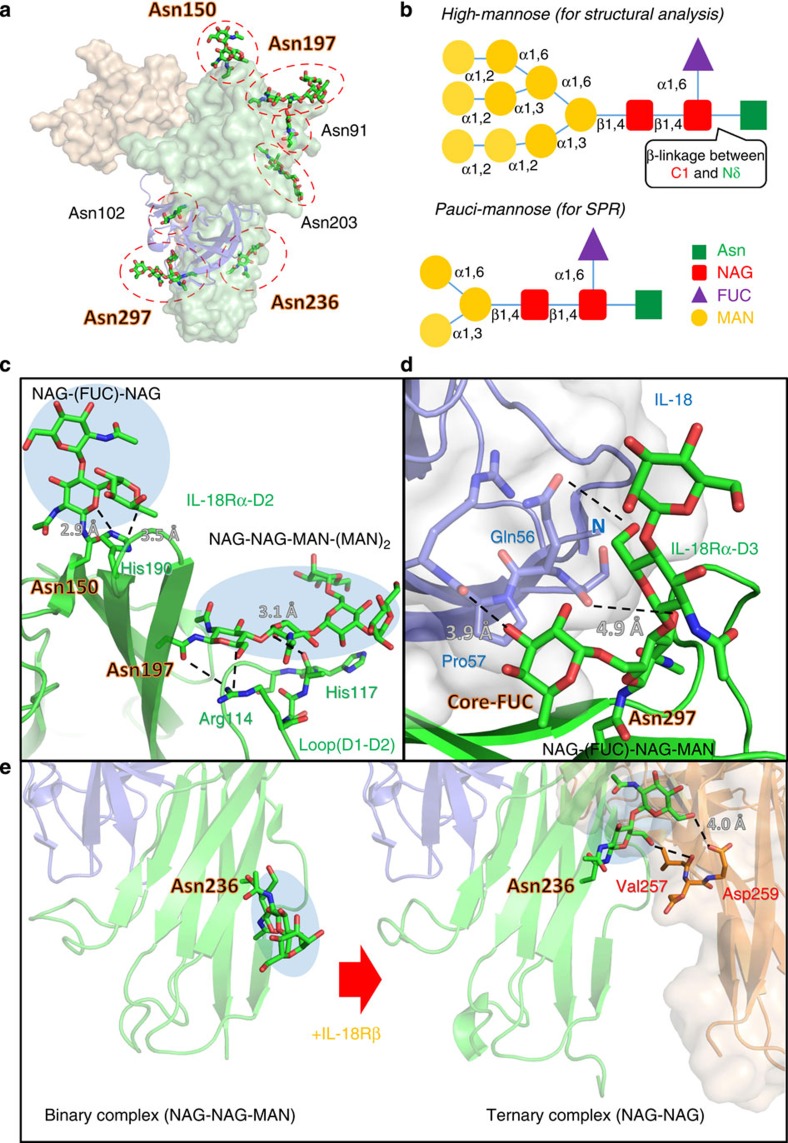
The sugar contacts that maintain IL-18/IL-18Rα/IL-18Rβ. (**a**) The N-linked glycans identified from the structure. (**b**) The N-linked glycosylation in this study. (**c**) Intramolecular interactions between the carbohydrates on Asn150^Rα^ and His190^Rα^ as well as on Asn197^Rα^ and the D1-D2 loop. (**d**) Interactions between the N-linked carbohydrates on Asn297^Rα^ and IL-18. Core-FUC moderately interacts with IL-18 at an ~4 Å distance. (**e**) Interactions between the N-linked carbohydrates on Asn236^Rα^ and IL-18Rβ.

In the IL-18/IL-18Rα and IL-18/IL-18Rα/IL-18Rβ crystal structures, seven N-linked glycosyl chains were identified in IL-18Rα at Asn91, 102, 150, 197, 203, 236 and 297 (Fig. 6a). These carbohydrates are high-mannose glycans (Fig. 6b) because the receptor proteins were prepared using the Sf9 expression system. The glycan on Asn197^Rα^, which are located in the D2 domain, forms moderate intramolecular interactions with Arg114^Rα^ and His117^Rα^ at the D1-D2 loop (Fig. 6c), seemingly contributing to the subdomains’ spatial arrangement. The core-NAG (N-acetyl-D-glucosamine) directly linked to Asn297^Rα^ branches into the second-NAG and L-fucose (FUC), which is referred to as the core-FUC (Fig. 6b–d). Interestingly, the core-FUC and second-NAG on Asn297^Rα^ are proximal to the β4-β5 loop of IL-18 (<4 Å); hence, they likely interact through electrostatic and hydrophobic interactions with the ligand (Fig. 6d) and partly contribute the unique D3:D3 interaction.

Notably, the NAG-NAG extended from the IL-18Rα-D3 Asn236^Rα^ and points to the receptor C-terminus in the binary complex; however, in the ternary complex, the NAG-NAG chain changes direction and points to IL-18Rβ-D3 within the distance possibly to form electrostatic interaction with Val257^Rβ^ and Asp259^Rβ^ (Fig. 6e). A similar inter-receptor interaction via an N-linked oligosaccharide was previously observed between IL-1RI-D3 and IL-1RAcP-D3 in the IL-1β signalling complex, but it is intriguingly absent in the IL-1β/IL-1RII/IL-1RAcP non-signalling decoy complex, which suggests that the N-linked oligosaccharides bridging the two receptors are important for IL-1 family signal transduction.

### Characterization of IL-18/IL-18Rα/IL-18Rβ in solution

**Figure 7:**
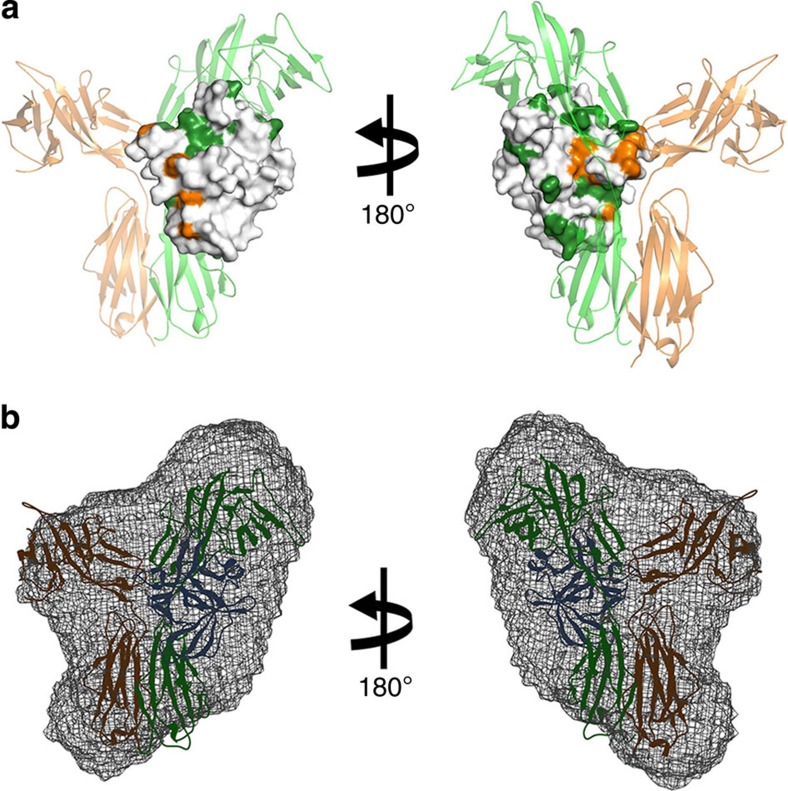
The solution-state IL-18/IL-18Rα/IL-18Rβ binding mode. (**a**) The results of cross-saturation experiments with [^2^H–^15^N]-IL-18/IL-18Rα (forest) and the chemical shift change of [^2^H–^15^N]-IL-18/IL-18Rα on adding IL-18Rβ (orange) are coloured on the crystal structure of IL-18 in complex with IL-18Rα and IL-18Rβ. (**b**) Superimposition of the IL-18/IL-18Rα/IL-18Rβ crystal structure and the low-resolution SAXS envelope.

A previous SPR analysis showed that IL-18Rβ does not bind free IL-18, but it does bind a preformed IL-18/IL-18Rα binary complex^19^. To better understand the formation of this complex under more physiological conditions, we performed titration experiments using solution NMR spectroscopy. The ^1^H–^15^N correlation spectrum for [^2^H,^15^N]-IL-18 did not change on adding a small excess of IL-18Rβ, indicating that the molecules do not interact. However, marked spectral changes were observed when IL-18 was titrated with IL-18Rα (Supplementary Fig. 4a,b). The amino-acid residues that were perturbed during the titration are at the molecular interface of the binary complex, which indicates that the binding mode in solution is identical to the crystal structure (Supplementary Fig. 4b, bottom). Cross-saturation NMR experiments^28^ further confirmed this result with more precision (Fig. 7a, forest and Supplementary Fig. 4c). Next, IL-18Rβ was added to the preformed [^2^H,^15^N]-IL-18/IL-18Rα binary complex. The ^1^H–^15^N correlation spectrum was then changed due to ternary complex formation. Although significant changes were not observed in the region including the previously defined Site III (Supplementary Fig. 4d), the cross-peaks that disappeared or shifted on ternary complex formation (Supplementary Fig. 4e) are mostly at the interface with IL-18Rβ, which is consistent with the ternary complex crystal structure (Fig. 7a, orange). Furthermore, we performed SAXS to analyse the architecture of the ternary complex (Supplementary Fig. 4f–h); these data were used to construct a low-resolution dummy atom model. The crystal structure of the complex reasonably fits the SAXS-derived model envelope (Fig. 7b). Together, the crystal structures solved in this study are consistent with the data collected in the solution state, which underscores the physiological significance of the crystal structures.

### Effects of mutation on the signaling and binding affinities

**Figure 8:**
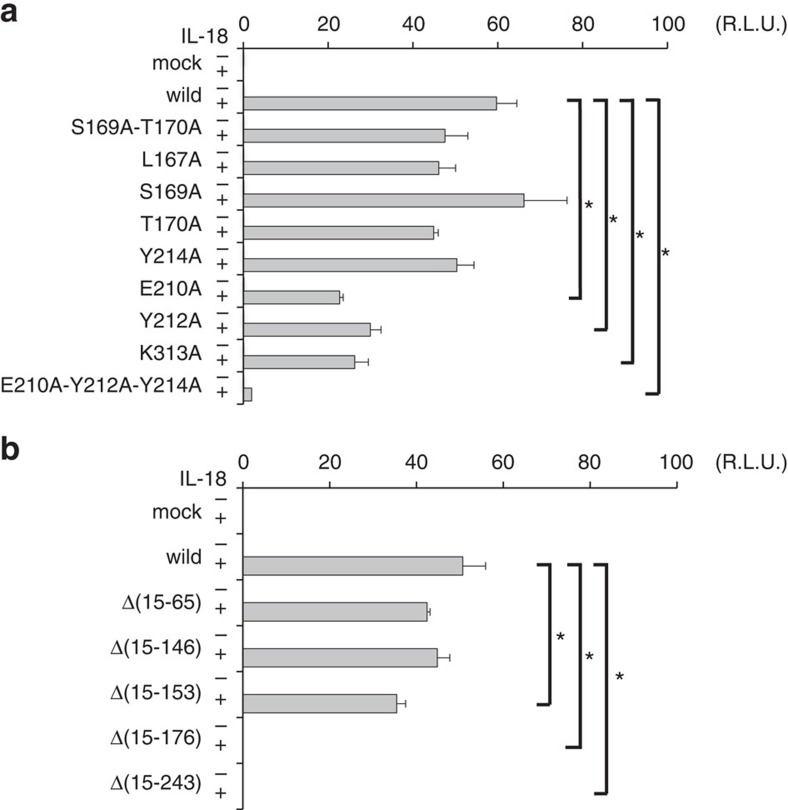
The effect of mutations on protein–protein interfaces for signal transduction. Cell-based NF-κB activity assay for IL-18Rβ with (**a**) an alanine substitution and (**b**) N-terminal deletion. Each column indicates the relative luciferase activity unit (R.L.U.), for the stimulated cells(+) and the non-stimulated cells(−). The data are the mean±s.d. for triplicate experiments. Asterisks inside the graphs indicate the significance from comparing mutant versus wild-type IL-18 Rβ, which was determined using a one-way ANOVA with Bonferroni’s multiple comparison test; *P<0.05. This experiment is representative of three independent experiments.

To confirm the importance of the IL-18 Site III interactions in signal transduction, we performed cell-based assays using IL-18Rβ mutants (Fig. 8a). IL-18Rβ-WT and its mutants were transiently expressed in HEK293 cells with a background of stably expressed IL-18Rα; the NF-κB activity was measured using the luciferase reporter system with or without an IL-18 stimulus. A complete loss of function was observed for the IL-18Rβ-E210A-Y212A-Y214A triple mutants, while IL-18Rβ-E210A, -Y212A and -K313A exhibited approximately half the activity compared with -WT. Remarkably, only a minor decrease in activity was observed when the D1 region of IL-18Rβ was deleted (Δ15–65 and Δ15–146, Fig. 8b), likely because IL-18Rβ-D1 is distal to the other parts of the ternary complex and seemingly does not affect binding. However, a substantial decrease in activity was observed when the D1-D2 loop (Δ15–153) was deleted, and the activity was fully abolished in the D1/D2-deficient experiments (Δ15–176 and Δ15–243). Therefore, IL-18Rβ-D2+D3 is sufficient, but D1 is not essential for signalling.

To identify the amino-acid residues that are critical for ternary complex formation, we performed a binding study using surface plasmon resonance (SPR) analysis (Supplementary Figs 5 and 6 and Supplementary Table 1). The prominent binding residues are summarized in Table 2 and Supplementary Fig. 7. First, IL-18Rβ was immobilized on the sensor chip, and binary complexes that formed between one of IL-18 mutants and IL-18Rα were examined for binding. When the binary complex contained either of three IL-18 mutants, G108A, H109A or K112A, it lost affinity for IL-18Rβ, even though these mutants maintained full binding activity for IL-18Rα. Thus, the mutated residues are only important for IL-18Rβ binding. Next, one of IL-18Rβ mutants was immobilized on the sensor chip, and the IL-18/IL-18Rα binary complex was examined for binding. Our data show that IL-18Rβ-Y212A did not bind the binary complex, which is consistent with the structure, wherein substantial π-π stacking were observed between Tyr212^Rβ^ and His109^IL-18^ (Fig. 5c). IL-18Rβ-K313A and -E210A exhibited 7- and 20-fold lower affinity for the IL-18/IL-18Rα complex relative to the wild-type receptor, respectively. These data are also consistent with the NF-κB luciferase reporter assay and the structure, wherein the mutated residues extensively interact with His109^IL-18^, Asp110^IL-18^, Lys112^IL-18^ and Phe135^Rα^ (Fig. 5c and Supplementary Fig. 3a).

To determine the contribution of the IL-18Rα N-linked oligosaccharides to the receptor complex formation, we mutated two IL-18Rα asparagine residues to glutamine (Table 2). The affinity of IL-18 for IL-18Rα decreased to one-third when Asn297^Rα^ was mutated to Gln compared with the wild type, which indicates that the sugar chain is important for the recognition of ligand, wherein the Asn297^Rα^ core-FUC and second-NAG are proximal to IL-18 (Fig. 6d). However, we did not observe a different affinity on mutating Asn236^Rα^.

## Discussion

It is well-known that the IL-1 family system has only two co-receptors, IL-1RAcP and IL-18Rβ, despite its seven agonists (IL-1α, IL-1β, IL-18, IL-33, IL-36α, IL-36β and IL-36γ) and four primary receptors (IL-1RI, IL-18Rα, ST2 and IL-36R)^29^. Thus, certain receptors must be used by multiple ligands (Fig. 2a). In fact, IL-1RAcP is commonly involved in signals with six ligands other than IL-18, which is recognized by its specific receptors, IL-18Rα and IL-18Rβ. Accordingly, the IL-18 binding mode remained unclear even after several crystal structures of the IL-1β and receptor complexes became available. The IL-18 complex structures determined in this work exhibited essentially the same binding mode as IL-1β^23^,^24^,^25^. This feature was confirmed under more physiological conditions by using two solution techniques, NMR and SAXS. On the basis of these results, it is highly likely that the left binding mode is the common mode in the IL-1 family; however, the IL-36/IL-36R/IL-1RAcP ternary complex structure has not been determined. Although the general binding mode was conserved, substantial differences were observed at the ligand-receptor or receptor-receptor interfaces in the IL-18 and IL-1β ternary complexes, which was expected based on the significant sequence deviations (Supplementary Fig. 1). The pronounced effects from such deviations include the unique compositions of secondary structure elements in IL-18Rα-D1 and IL-18Rs-D2, such as the lack of a β-strand that is typically conserved among Ig-like domains. This feature has led to the unique subdomain orientation of IL-18Rβ, and may partly explain why IL-1RAcP exhibits the promiscuous ligand recognition mode, although IL-18Rβ does not. On the basis of the presumable existence of d2 in IL-36R-D2 and the IL-36 (ref. 27) structure, it is likely that the IL-36 system also adopts the left binding mode with similar orientation to the IL-1β system.

Furthermore, we found a potential common trait among the IL-1 family, which is that the N-linked glycans presumably bridge two receptors in the ternary complex. Intriguingly, this feature was only observed in two functional ternary complexes (IL-1β/IL-1RI/IL-1RAcP and IL-18/IL-18Rα/IL-18Rβ) but was absent in the decoy ternary complex (IL-1β/IL-1RII/IL-1RAcP) and may contribute the binding affinity. Nevertheless, our SPR analyses showed that the difference in IL-18Rα-binding affinity for IL-18Rβ was only trivial even without glycosylation (N236Q^Rα^ in Table 2). This discrepancy may be due to the distinct sugar modification patterns between insects and mammals. N-glycans are mostly pauci-mannose (Fig. 6b, not greater than three MAN) oligosaccharides in silkworm^30^, while mammals contain more varied outer sugar chains; certain chains are longer with more complicatedly mixtures (that is, MAN, NAG, galactose and sialic acid). Thus, larger interaction surfaces on N236^Rα^ sugar chains in humans are expected, which could strengthen the affinity. In contrast, one N-glycan chain likely plays a unique role in the IL-18 system, as the IL-18Rα Asn297^Rα^ sugar chain moderately interacts with IL-18 in both the binary and ternary complexes (Fig. 6d). In fact, IL-18Rα without the sugar chain (N297Q) exhibited a threefold lower binding affinity (Table 2). To our best knowledge, such sugar–ligand interactions have not been reported for other IL-1 family members. For the IL-1β complexes, any N-glycan chains on IL-1RI(RII) appear too distal to the ligand for direct contact.

IL-1 family activity *in vivo* are modulated by the counteractions of natural inhibitors, such as receptor antagonists (IL-1Ra, IL-36Ra and IL-38), soluble receptors (sIL-1RI, sIL-18R and sST2) and IL-18BP. These proteins tightly bind their target cytokines or receptors, which impedes formation of ternary complexes that initiate intracellular signal transduction. This balancing feature facilitates secure control of inflammatory responses *in vivo*. Our structures not only demonstrate the receptors’ molecular recognition mode for IL-18, but they also explain the IL-18 inhibitors’ mechanism of action. Superimposition of the IL-18/IL-18Rα and IL-18/IL-18BPs complex structures^20^,^22^ shows that the IL-18BP binding site on IL-18 precisely corresponds with the IL-18Rα-D3 binding site. Thus, IL-18BP clearly inhibits IL-18Rα binding through steric hindrance (Supplementary Fig. 8a). Recently, IL-37 (IL-1F7b) was proposed to function as an anti-inflammatory cytokine^31^. IL-37 weakly bound IL-18Rα with 50 times lower affinity than IL-18 (Fig. 2a), and did not activate signal transduction^32^. Consistent with this notion, the essential IL-18 amino acids for the IL-18Rβ interactions, as well as for IL-18Rα, determined in this study are not conserved in IL-37 (Supplementary Fig. 1a), which suggests that IL-37 is incapable of forming the ternary complex that enables signal transduction. In addition to the natural inhibitors, several anti-IL-18 antibodies have been developed for therapeutic purposes^21^,^33^. The structures herein clearly explain how these antibodies impede binding between the ligand and receptors (Supplementary Fig. 8b).

The molecular interface differences herein are important because they may provide numerous opportunities for designing molecules that specifically stabilize or interrupt IL-18 ternary complex formation without affecting other IL-1 family members. Unlike molecules that target more downstream signalling pathways, these molecules can be used to treat ligand-specific diseases and avoid side effects due to interference with common intracellular signalling pathways. The structural information collected in this work will facilitate development of an IL-18 modulator in multiple ways. For example, recombinant derivatives of IL-18, which bind to IL-18Rα more strongly relative to wild type of IL-18, can be logically designed based on the structures^34^. The structures will especially benefit small molecule design; small molecules are generally more cost effective once developed, and thus preferable to proteinous compounds. The data obtained in this study provide an atomic framework for molecular interfaces between IL-18 and receptors as well as between the two receptors, which serve as promising drug target sites, and hence will aid in development of effective IL-18 inhibitors.

In summary, the structures of IL-18 and its receptor have advanced our precise understanding of molecular recognition and suggest a single architectural paradigm for signalling complexes in the IL-1 family cytokines. Furthermore, with biochemical and cellular data, the structures reveal detailed interaction properties at the molecular interfaces, presenting an atomic framework that will aid in rational drug development for IL-18-related diseases.

## Methods

### Construction of expression vectors

The coding region of the extracellular domains of human IL-18Rα (NM_003855, residues 20–329) and IL-18Rβ (NM_003853, residues 15–356) were cloned into the pFastBac1 vector (Invitrogen, Carlsbad, CA, USA). Full-length IL-18Rβ was also cloned into the pcDNA3.1+ vector (Invitrogen). The pGL4.32[luc2P/NF-κB-RE/Hygro] vector was used as an NF-κB luciferase reporter, and the pGL4.70[hRluc] vector was used as an internal control *Renilla* luciferase reporter; both were purchased from Promega (Fitchburg, WI, USA). For the crystallographic studies, the signal-peptide sequence for Sf9 insect cells, an 8 × His tag and an HRV 3C protease cleavage site were placed immediately upstream of the mature sequence^35^. The same constructs with a C-terminal 6 × His tag and without the HRV 3C site were also prepared for solution structure analysis and SPR experiments. Mutations were introduced into the pGEX4T-1[IL-18]^19^, pFastBac1[IL-18Rs] and pcDNA3.1+[IL-18Rβ] vectors using an inverse PCR-based site-directed mutagenesis method.

### Protein expression and purification

Mature IL-18 (residues 1–157) and its single amino-acid mutants were prepared as previously reported^19^. In brief, human IL-18 was expressed as a glutathione S-transferase (GST) fusion protein in the *Escherichia coli* strain BL21(DE3) (Novagen, Madison, WI, USA). GST-tagged IL-18 was affinity purified followed by GST digestion with Factor Xa and further purified using gel filtration column chromatography. Expression of IL-18Rs using Sf9 insect cell system and their detailed purification procedures were also described^35^. The extracellular human IL-18Rα or IL-18Rβ domains were each separately secreted from Sf9 insect cells (Invitrogen) for structural analyses. The supernatant was purified through three chromatography steps, including ion exchange chromatography, Ni-NTA affinity chromatography and gel filtration chromatography with or without an HRV 3C treatment. To obtain the IL-18/IL-18Rα binary and IL-18/IL-18Rα/IL-18Rβ ternary complexes, IL-18, IL-18Rα and IL-18Rβ were mixed at equimolar ratios and purified through gel filtration chromatography. Both the wild type and the mutants of the IL-18Rs for SPR analysis were expressed using a silkworm system^36^,^37^. The donor plasmids of the pFastBac1 vectors containing the IL-18 receptor gene were transformed into *Escherichia coli* BmDH10Bac. Then, 1 μg of BmNPV bacmid DNA and 1 μl of Cellfectin reagent (Invitrogen) suspended in Grace insect cell medium were injected into the ventral side of *B. mori* silkworm larvae. After 6 days, haemolymph was recovered from the larvae, and sodium thiosulfate (final 0.5%) and EDTA were immediately added. The IL-18 receptor proteins from the silkworm were purified using the same protocols as the purification from the Sf9 insect cell system.

### Cell culture

HEK293 cells (Japanese Collection of Research Bioresources, Osaka, Japan) were cultured in Dulbecco’s modified Eagle’s medium (high glucose-containing D-MEM, Invitrogen) supplemented with 10% heat-inactivated fetal bovine serum (Sigma-Aldrich, Missouri, USA), penicillin (100 unit ml^−1^) and streptomycin (100 μg ml^−1^). All cells were incubated at 37 °C in a humidified atmosphere of 5% CO_2_.

### Luciferase reporter gene assay

HEK293 cells were transfected with an empty pcDNA3.1+ vector or pcDNA3.1+[IL-18Rβ] (wild-type or mutants), pGL4.32[luc2P/NF-κB-RE/Hygro] and pGL4.70[hRluc] vectors using Lipofectamine 2000 reagent according to the manufacturer’s instructions. These transfectants were stimulated with recombinant human IL-18 (10 ng ml^−1^) for 6 h. The luciferase reporter gene activities were analysed using a Dual-Luciferase Reporter Assay System (Promega). The statistical significance of the differences was determined using one-way ANOVA with Bonferroni’s multiple comparison test. The statistical significance was assigned to be *P<*0.05.

### Surface plasmon resonance analysis

The real-time binding affinities between IL-18 and IL-18Rα and between IL-18Rβ and IL-18/IL-18Rα were analysed using a BIAcore 3000 surface plasmon resonance spectrometer (GE Healthcare, Little Chalfont, UK) at 25 °C with a Ni-NTA sensor chip. The *K*_d_ (dissociation constant) estimated using the Ni-NTA sensor chip was an average of one order of magnitude lower than estimates obtained using an Anti-His-tag Ab covalently linked to a CM5 sensor chip. However, we used the Ni-NTA sensor chip because it did not decrease the binding capacity after repeated measurement cycles, which is desirable when comparing the *K*_d_ of many mutants.

C-terminal 6 × His tagged IL-18Rα was immobilized approximately 200-resonance units (RU) on an NTA sensor chip. Then, various concentrations of IL-18 in HBS-P (10 mM HEPES-Na, pH7.4, 150 mM NaCl, 0.01% (v/v) surfactant P-20) buffer were injected over the sensor surface as an analyte at a flow rate of 30 μl min^−1^ for 180 s. After association, it was allowed to run for another 360 s for dissociation. In the same way, C-terminal 6 × His tagged IL-18Rβ was immobilized approximately 100 RU on the sensor chip, and un-tagged IL-18Rα that was saturated with 500 nM IL-18 in HBS-P buffer was injected. The sensor surface was regenerated with 350 mM EDTA. For the mutational analysis, mutants of IL-18, IL-18Rα and IL-18Rβ were used instead of wild type, as shown in Supplementary Table 1. The sensor chip was analysed using BIAevaluation software (GE Healthcare). Analyses with the same concentration series were performed in triplicate.

### Crystal structure determination

Step by step crystallization method and preliminary crystallographic analysis were done as described^35^. X-ray diffraction data were collected at 100 K on the BL38 (IL-18) or BL44XU (IL-18/IL-18Rα) beamlines at SPring-8 (Harima, Japan) and on the BL17A beamline (IL-18/IL-18Rα/IL-18Rβ) at Photon Factory (Tsukuba, Japan). For IL-18 and IL-18/IL-18Rα, the diffraction data were processed using the XDS^38^ and SCALA^39^,^40^ software. The initial phases were determined by molecular replacement using Phaser^41^ with the crystal structure of IL-18 (ref. 20) (PDB code: 3F62) as the search model. The initial phases of the binary complex were improved by NCS averaging using RESOLVE^42^. The model was further manually built using COOT^43^ and refined using BUSTER^44^ with autoNCS^45^. In the IL-18 and IL-18/IL-18Rα crystals, four and six copies of IL-18 and the binary complexes were observed in each ASU, respectively. The structures of these copies in the ASUs are essentially the same, so we referred to chain A for IL-18 and chain A/B for IL-18/IL-18Rα, if not otherwise indicated. The structure of IL-18 included 13 molecules of CHAPS. The diffraction data of IL-18/IL-18Rα/IL-18Rβ were processed using HKL2000 software^46^. To determine the initial phase for IL-18/IL-18Rα/IL-18Rβ, a refined structure of the binary complex was used as the search model for molecular replacement. The model was further manually built using COOT and refined using BUSTER. Only one copy of the ternary complex was in the IL-18/IL-18Rα/IL-18Rβ crystal ASU. Ramachandran diagrams were examined using RAMPAGE^47^. Structural figures were prepared using PyMol (Schrödinger, LLC). The secondary structures were assigned using the DSSP software^48^.

### NMR spectroscopy

All NMR spectra were measured at 308 K on a Bruker Avance II 700 MHz spectrometer equipped with cryogenic probes using TROSY-type pulse sequences. The samples for NMR measurements were in 20 mM potassium phosphate (pH 6.0), 50 mM KCl in H_2_O/D_2_O (90%/10%). Spectra were processed using NMRPipe^49^ and analysed using Sparky analysis software^50^.

Chemical shift assignments for IL-18 bound to IL-18Rα are based on a HN(CO)CA/HNCA data set and confirmed by HNCACB and NOESY spectra with a mixing time of 200 ms. The IL-18Rα binding surface of IL-18 in solution is defined by the cross-saturation^28^ intensity ratio of I_2000ms_/I_0ms_. The IL-18Rβ binding site of IL-18 is inferred from the titration experiment, by shifted or eliminated peaks, which can be assigned for all but the IL-18Rα binding surface. Structural figures were prepared using PyMol (Schrödinger, LLC).

### Small-angle X-ray scattering

SAXS data of the IL-18/IL-18Rα/IL-18Rβ ternary complex were collected at the beamline 12ID-B of the Advanced Photon Source at Argonne National Laboratory (Argonne, IL, USA). The sample concentrations were 1, 3 and 5 mg^−1^ ml^−1^. The samples were run at 12 keV radiation energy, with a sample-to-detector distance of 2 m. The scattered X-rays were measured using a Pilatus 2 M detector. A flow cell was used to reduce radiation damage. Thirty images were taken for each blank and each sample.

After background subtraction, the data were superimposed using Primus^51^ (Supplementary Fig. 4f). *S* is the momentum transfer equal to 4πsin(*θ*/2)/*λ*, where *θ* and *λ* are the scattering angle and X-ray wavelength, respectively. *R*_g_ is the radius of gyration, which was determined using the Guinier approximation of the data in the low *s* region (*sR*_g_<1.3), the linearity of which also served as an initial assessment of data quality (Supplementary Fig. 4g). The maximum particle dimension, *D*_max_, and the distance distribution function, *P*(*r*), were calculated using auto GNOM^52^ (Supplementary Fig. 4h). The low-resolution envelopes of the ternary complex were produced using DAMMIN^53^ by directly fitting the reciprocal space scattering profile. Fifteen DAMMIN models were generated and then aligned and averaged using DAMAVER and DAMFILT^54^. Structural figures were prepared using Chimera^55^.

## Author contributions

H.T. and Z.K. designed the work; M.S and N.K. supervised the study; N.T. carried out the experimental design, experiments and structural analyses, supported by T.K., K.A., M.A. and H.T.; T.K. prepared the samples for the study, supported by H.O., N.T., K.M. and E.Y.P.; T.K. performed and analysed the SPR experiments; T.Y. performed the cell assays, supported by H.O. and T.K.; X.Z. performed the SAXS measurements; and N.T., T.K., K.A., H.O., Z.K. and H.T. contributed to writing the manuscripts and preparing the figures.

## Additional information

**How to cite this article**: Tsutsumi, N. *et al.* The structural basis for receptor recognition of human interleukin-18. *Nat. Commun.* 5:5340 doi: 10.1038/ncomms6340 (2014).

**Accession codes**: The atomic coordinates and structure factors for IL-18, IL-18/IL-18Rα and IL-18/IL-18Rα/IL-18Rβ have been deposited in the RCSB Protein Data Bank under accession codes 3WO2, 3WO3 and 3WO4, respectively.

## Supplementary Material

Supplementary Information Supplementary Figures 1-8, Supplementary Table 1 and Supplementary References

## Figures and Tables

**Table 1:** X-ray crystallographic statistics of IL-18 and its extracellular complexes.

	**IL-18**	**IL-18/IL-18Rα**	**IL-18/IL-18Rα/IL-18Rβ**
*Data collection*			
Beamline	BL38B1 (SPring-8)	BL44XU (SPring-8)	BL17A (Photon Factory)
Wavelength	1.0000	0.9000	0.9800
Space group	*P2*_*1*_	*P2*_*1*_*2*_*1*_*2*	*P2*_*1*_*2*_*1*_*2*_*1*_
Cell dimensions			
*a*, *b*, *c* (Å)	68.15, 79.51, 73.46	135.49, 174.81, 183.40	72.56, 111.56, 134.57
α, β, γ (°)	90.00, 100.97, 90.00	90.00, 90.00, 90.00	90.00, 90.00, 90.00
Resolution (Å)	45.0–2.33 (2.46–2.33)	43.9–3.10 (3.27–3.10)	50.0–3.10 (3.21–3.10)
*R*_merge_	4.9 (39.6)	10.0 (58.2)	11.5 (68.3)
*I*/σ*I*	20.5 (3.6)	14.9 (2.5)	19.8 (2.9)
Completeness (%)	97.8 (97.1)	99.6 (100)	99.9 (99.3)
Redundancy	3.8 (3.8)	3.7 (3.8)	9.8 (9.2)
			
*Refinement*			
Resolution (Å)	34.82–2.33 (2.41–2.33)	42.00–3.10 (3.18–3.10)	42.00–3.10 (3.29–3.10)
No. reflections	32,157 (2,861)	79,130 (5,821)	17,368 (1,825)
*R*_work_/*R*_free_	21.8/27.1 (27.6/29.8)	19.1/22.2 (22.7/26.3)	18.8/23.2 (20.9/29.7)
No. atoms			
Protein	5,022	20,533	5,663
Glycan	—	1,225	335
Others	711	289	32
*B*-factors			
Protein	46.13	78.31	74.38
Glycan	—	121.43	139.74
Others	60.94	109.02	36.55
R.m.s. deviations			
Bond lengths (Å)	0.010	0.009	0.009
Bond angles (°)	1.38	1.11	1.17
Ramachandran plot	95.2, 4.8	94.4, 5.0, 0.5	93.7, 5.4, 0.8

Values for Ramachandran plots are presented as favoured, allowed, outlier examined by RAMPAGE. Data collection statistics were summarized from the report^35^.

**Table 2:** Functional epitopes of IL-18 and IL-18 receptors.

	***K***_**d**_ **(10**^**−8**^ **M)**
Ligand: IL-18Rβ WTAnalyte: IL-18 mutant/IL-18Rα WT complex	
IL-18 WT(control)	1.5±0.1
IL-18 G108A	No binding
IL-18 H109A	No binding
IL-18 D110A	5.6±0.8
IL-18 K112A	No binding
IL-18 G145A	2.8±0.1
IL-18 R147A	5.7±0.3
IL-18 M150A	6.0±0.5
	
Ligand: IL-18Rβ mutantAnalyte: IL-18 WT/IL-18Rα WT complex	
IL-18Rβ WT (control)	1.5±0.1
IL-18Rβ L167A	3.3±0.4
IL-18Rβ E210A	32.7±0.9
IL-18Rβ Y212A	No binding
IL-18Rβ Y214A	5.0±0.5
IL-18Rβ K313A	10.9±0.9
	
Ligand: IL-18Rβ WTAnalyte: IL-18WT/IL-18Rα mutant complex	
IL-18Rα WT (control)	1.5±0.1
IL-18Rα N236Q	1.7±0.3
	
Ligand: IL-18Rα mutantAnalyte: IL-18 WT	
IL-18Rα WT (control)	6.9±0.2
IL-18Rα N297Q	25.8±2.6

Ligand: the interactant captured on the sensor surface. Analyte: the interactant in free solution. WT: wild type. Values are the means±s.d. of the results derived from three independent experiments.

## References

[b1] Okamura H. *et al.* Cloning of a new cytokine that induces IFN-gamma production by T cells. Nature 378, 88–91 (1995). 7477296 10.1038/378088a0

[b2] Kuida K. *et al.* Altered cytokine export and apoptosis in mice deficient in interleukin-1 beta converting enzyme. Science 267, 2000–2003 (1995). 7535475 10.1126/science.7535475

[b3] Li P. *et al.* Mice deficient in IL-1 beta-converting enzyme are defective in production of mature IL-1 beta and resistant to endotoxic shock. Cell 80, 401–411 (1995). 7859282 10.1016/0092-8674(95)90490-5

[b4] Ghayur T. *et al.* Caspase-1 processes IFN-gamma-inducing factor and regulates LPS-induced IFN-gamma production. Nature 386, 619–623 (1997). 9121587 10.1038/386619a0

[b5] Rathinam V. a. K., Vanaja S. K. & Fitzgerald K. a. Regulation of inflammasome signaling. Nat. Immunol. 13, 333–2 (2012). 22430786 10.1038/ni.2237 PMC3523703

[b6] Ohnishi H. *et al.* TRAM is involved in IL-18 signaling and functions as a sorting adaptor for MyD88. PLoS ONE 7, e38423 (2012). 22685567 10.1371/journal.pone.0038423 PMC3369926

[b7] Adachi O. *et al.* Targeted disruption of the MyD88 gene results in loss of IL-1- and IL-18-mediated function. Immunity 9, 143–150 (1998). 9697844 10.1016/s1074-7613(00)80596-8

[b8] Hoffman H. M., Mueller J. L., Broide D. H., Wanderer A. A. & Kolodner R. D. Mutation of a new gene encoding a putative pyrin-like protein causes familial cold autoinflammatory syndrome and Muckle-Wells syndrome. Nat. Genet. 29, 301–305 (2001). 11687797 10.1038/ng756 PMC4322000

[b9] Park H., Bourla A. B., Kastner D. L., Colbert R. a. & Siegel R. M. Lighting the fires within: the cell biology of autoinflammatory diseases. Nat. Rev. Immunol. 12, 570–580 (2012). 22828911 10.1038/nri3261 PMC4165575

[b10] Dinarello C. a., Simon A. & van der Meer J. W. M. Treating inflammation by blocking interleukin-1 in a broad spectrum of diseases. Nat. Rev. Drug Discov. 11, 633–652 (2012). 22850787 10.1038/nrd3800 PMC3644509

[b11] Alboni S., Cervia D., Sugama S. & Conti B. Interleukin 18 in the CNS. J. Neuroinflamm. 7, 9 (2010). 10.1186/1742-2094-7-9 PMC2830964 20113500

[b12] Mellins E. D., Macaubas C. & Grom A. A. Pathogenesis of systemic juvenile idiopathic arthritis: some answers, more questions. Nat. Rev. Rheumatol. 7, 416–426 (2011). 21647204 10.1038/nrrheum.2011.68 PMC4180659

[b13] Hirota T. *et al.* Genome-wide association study identifies eight new susceptibility loci for atopic dermatitis in the Japanese population. Nat. Genet. 44, 1222–1226 (2012). 23042114 10.1038/ng.2438

[b14] Dinarello C. A., Novick D., Kim S. & Kaplanski G. Interleukin-18 and IL-18 binding protein. Front. Immunol. 4, 289 (2013). 24115947 10.3389/fimmu.2013.00289 PMC3792554

[b15] Romberg N. *et al.* Mutation of NLRC4 causes a syndrome of enterocolitis and autoinflammation. Nat. Genet. 46, 1135–1139 (2014). 25217960 10.1038/ng.3066 PMC4177367

[b16] Canna S. W. *et al.* An activating NLRC4 inflammasome mutation causes autoinflammation with recurrent macrophage activation syndrome. Nat. Genet. 46, 1140–1146 (2014). 25217959 10.1038/ng.3089 PMC4177369

[b17] Terada M. *et al.* Contribution of IL-18 to atopic-dermatitis-like skin inflammation induced by Staphylococcus aureus product in mice. Proc. Natl Acad. Sci. USA 103, 8816–8821 (2006). 16723395 10.1073/pnas.0602900103 PMC1482661

[b18] Brydges S. D. *et al.* Divergence of IL-1, IL-18, and cell death in NLRP3 inflammasomopathies. J. Clin. Invest. 123, 4695–4705 (2013). 24084736 10.1172/JCI71543 PMC3809806

[b19] Kato Z. *et al.* The structure and binding mode of interleukin-18. Nat. Struct. Biol. 10, 966–971 (2003). 14528293 10.1038/nsb993

[b20] Krumm B., Meng X., Li Y., Xiang Y. & Deng J. Structural basis for antagonism of human interleukin 18 by poxvirus interleukin 18-binding protein. Proc. Natl Acad. Sci. USA 105, 20711–20715 (2008). 19104048 10.1073/pnas.0809086106 PMC2634891

[b21] Argiriadi M. A., Xiang T., Wu C., Ghayur T. & Borhani D. W. Unusual water-mediated antigenic recognition of the proinflammatory cytokine interleukin-18. J. Biol. Chem. 284, 24478–24489 (2009). 19553661 10.1074/jbc.M109.023887 PMC2782040

[b22] Krumm B., Meng X., Wang Z., Xiang Y. & Deng J. A unique bivalent binding and inhibition mechanism by the yatapoxvirus interleukin 18 binding protein. PLoS Pathog. 8, e1002876 (2012). 22927815 10.1371/journal.ppat.1002876 PMC3426546

[b23] Wang D. *et al.* Structural insights into the assembly and activation of IL-1β with its receptors. Nat. Immunol. 11, 905–911 (2010). 20802483 10.1038/ni.1925

[b24] Thomas C., Bazan J. F. & Garcia K. C. Structure of the activating IL-1 receptor signaling complex. Nat. Struct. Mol. Biol. 19, 455–457 (2012). 22426547 10.1038/nsmb.2260 PMC4006550

[b25] Liu X. *et al.* Structural insights into the interaction of IL-33 with its receptors. Proc. Natl. Acad. Sci. USA 110, 14918–14923 (2013). 23980170 10.1073/pnas.1308651110 PMC3773798

[b26] Vigers G. P., Anderson L. J., Caffes P. & Brandhuber B. J. Crystal structure of the type-I interleukin-1 receptor complexed with interleukin-1beta. Nature 386, 190–194 (1997). 9062193 10.1038/386190a0

[b27] Günther S. & Sundberg E. J. Molecular determinants of agonist and antagonist signaling through the IL-36 receptor. J. Immunol. 193, 921–930 (2014). 24935927 10.4049/jimmunol.1400538

[b28] Takahashi H., Nakanishi T., Kami K., Arata Y. & Shimada I. A novel NMR method for determining the interfaces of large protein-protein complexes. Nat. Struct. Biol. 7, 220–223 (2000). 10700281 10.1038/73331

[b29] Garlanda C., Dinarello C. a. & Mantovani A. The interleukin-1 family: back to the future. Immunity 39, 1003–1018 (2013). 24332029 10.1016/j.immuni.2013.11.010 PMC3933951

[b30] Park E. Y. *et al.* Human IgG1 expression in silkworm larval hemolymph using BmNPV bacmids and its N-linked glycan structure. J. Biotechnol. 139, 108–114 (2009). 18984019 10.1016/j.jbiotec.2008.09.013

[b31] Nold M. F. *et al.* IL-37 is a fundamental inhibitor of innate immunity. Nat. Immunol. 11, 1014–1022 (2010). 20935647 10.1038/ni.1944 PMC3537119

[b32] Kumar S. *et al.* Interleukin-1F7B (IL-1H4/IL-1F7) is processed by caspase-1 and mature IL-1F7B binds to the IL-18 receptor but does not induce IFN-gamma production. Cytokine 18, 61–71 (2002). 12096920 10.1006/cyto.2002.0873

[b33] Hamasaki T. *et al.* Human anti-human IL-18 antibody recognizing the IL-18-binding site 3 with IL-18 signaling blocking activity. J. Biochem. 138, 433–442 (2005). 16272137 10.1093/jb/mvi148

[b34] Kim S. H. *et al.* Site-specific mutations in the mature form of human IL-18 with enhanced biological activity and decreased neutralization by IL-18 binding protein. Proc. Natl Acad. Sci. USA 98, 3304–3309 (2001). 11248074 10.1073/pnas.051634098 PMC30649

[b35] Kimura T. *et al.* Purification, crystallization and preliminary X-ray crystallographic analysis of human IL-18 and its extracellular complexes. Acta Crystallogr. Sect. F, Struct. Biol. Commun. 70, 1351–1356 (2014). 25286938 10.1107/S2053230X14016926 PMC4188078

[b36] Motohashi T., Shimojima T., Fukagawa T., Maenaka K. & Park E. Y. Efficient large-scale protein production of larvae and pupae of silkworm by Bombyx mori nuclear polyhedrosis virus bacmid system. Biochem. Biophys. Res. Commun. 326, 564–569 (2005). 15596136 10.1016/j.bbrc.2004.11.060

[b37] Kajikawa M. *et al.* Silkworm Baculovirus Expression System for Molecular Medicine. J. Biotechnol. Biomater. S9, 1–5 (2012).

[b38] Kabsch W. Xds.. Acta Crystallogr. D. Biol. Crystallogr. 66, 125–132 (2010). 20124692 10.1107/S0907444909047337 PMC2815665

[b39] Evans P. Scaling and assessment of data quality. Acta Crystallogr. D. Biol. Crystallogr. 62, 72–82 (2006). 16369096 10.1107/S0907444905036693

[b40] Evans P. R. An introduction to data reduction: space-group determination, scaling and intensity statistics. Acta Crystallogr. D. Biol. Crystallogr. 67, 282–292 (2011). 21460446 10.1107/S090744491003982X PMC3069743

[b41] McCoy A. J. *et al.* Phaser crystallographic software. J. Appl. Crystallogr. 40, 658–674 (2007). 19461840 10.1107/S0021889807021206 PMC2483472

[b42] Terwilliger T. C. Statistical density modification with non-crystallographic symmetry. Acta Crystallogr. D. Biol. Crystallogr. 58, 2082–2086 (2002). 12454468 10.1107/S0907444902016360 PMC2745884

[b43] Emsley P. & Cowtan K. Coot: model-building tools for molecular graphics. Acta Crystallogr. D. Biol. Crystallogr. 60, 2126–2132 (2004). 15572765 10.1107/S0907444904019158

[b44] Bricogne G. *et al.* BUSTER. 2.10.0 Ed., Global Phasing Ltd, UK (2011).

[b45] Smart O. S. *et al.* Exploiting structure similarity in refinement: automated NCS and target-structure restraints in BUSTER. Acta Crystallogr. D. Biol. Crystallogr. 68, 368–380 (2012). 22505257 10.1107/S0907444911056058 PMC3322596

[b46] Otwinowski Z. & Minor W. Processing of X-ray diffraction data collected in oscillation mode. Methods Enzymol. 276, 307–326 (1997). 10.1016/S0076-6879(97)76066-X 27754618

[b47] Lovell S. C. *et al.* Structure validation by Calpha geometry: phi,psi and Cbeta deviation. Proteins 50, 437–450 (2003). 12557186 10.1002/prot.10286

[b48] Joosten R. P. *et al.* A series of PDB related databases for everyday needs. Nucleic Acids Res. 39, D411–D419 (2011). 21071423 10.1093/nar/gkq1105 PMC3013697

[b49] Delaglio F. *et al.* NMRPipe: a multidimensional spectral processing system based on UNIX pipes. J. Biomol. NMR 6, 277–293 (1995). 8520220 10.1007/BF00197809

[b50] Lee W., Westler W. M., Bahrami A., Eghbalnia H. R. & Markley J. L. PINE-SPARKY: graphical interface for evaluating automated probabilistic peak assignments in protein NMR spectroscopy. Bioinformatics 25, 2085–2087 (2009). 19497931 10.1093/bioinformatics/btp345 PMC2723000

[b51] Konarev P. V., Volkov V. V., Sokolova A. V., Koch M. H. J. & Svergun D. I. PRIMUS: a Windows PC-based system for small-angle scattering data analysis. J. Appl. Crystallogr. 36, 1277–1282 (2003).

[b52] Svergun D. I. Determination of the regularization parameter in indirect-transform methods using perceptual criteria. J. Appl. Crystallogr. 25, 495–503 (1992).

[b53] Svergun D. I. Restoring low resolution structure of biological macromolecules from solution scattering using simulated annealing. Biophys. J. 76, 2879–2886 (1999). 10354416 10.1016/S0006-3495(99)77443-6 PMC1300260

[b54] Volkov V. V. & Svergun D. I. Uniqueness of ab initio shape determination in small-angle scattering. J. Appl. Crystallogr. 36, 860–864 (2003). 10.1107/S0021889809000338 PMC5023043 27630371

[b55] Pettersen E. F. *et al.* UCSF Chimera-a visualization system for exploratory research and analysis. J. Comput. Chem. 25, 1605–1612 (2004). 15264254 10.1002/jcc.20084

