# Supplementary material for: The structural basis for receptor recognition of human interleukin-18
Source: Nat Commun. 2014 Dec 15;5:5340. doi: 10.1038/ncomms6340 (PMC4275594; doi:10.1038/ncomms6340)
Supplement: Supplementary Information — Supplementary Figures 1-8, Supplementary Table 1 and Supplementary References [file ncomms6340-s1.pdf]

Supplementary Information

The structural basis for receptor recognition of human interleukin-18

Naotaka Tsutsumi, Takeshi Kimura, Kyohei Arita, Mariko Ariyoshi, Hidenori Ohnishi, Takahiro Yamamoto, Xiaobing Zuo, Katsumi Maenaka, Enoch Y. Park, Naomi Kondo, Masahiro Shirakawa, Hidehito Tochio and Zenichiro Kato

Please contact H. O. (ohnishih@gifu-u.ac.jp) or H. T. (tochio@mb.biophys.kyoto-u.ac.jp)

SUPPLEMENTARY FIGURES

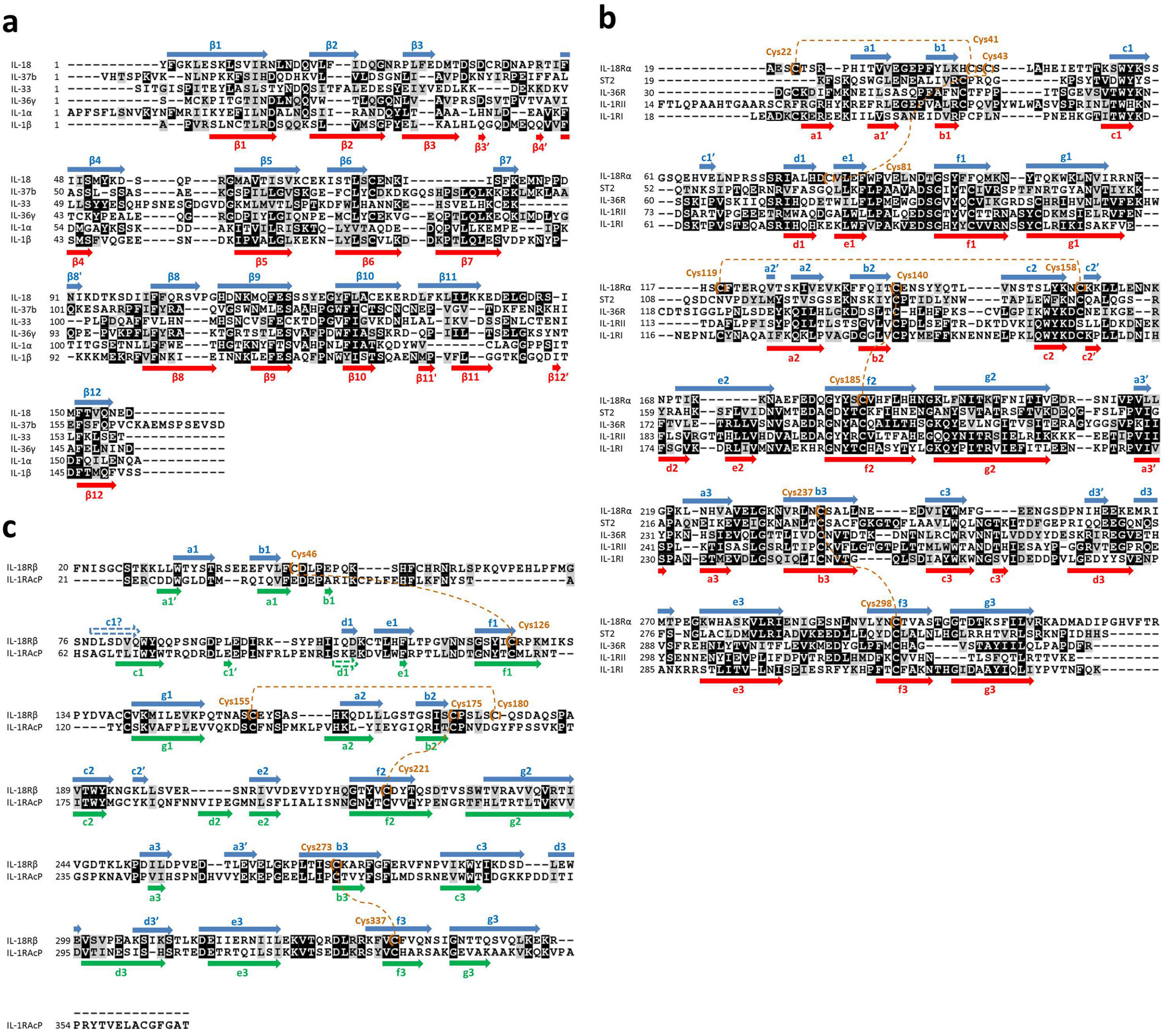

Supplementary Figure 1. Multiple sequence alignments for the IL-1 family system.

(a) The IL-1 family cytokines as well as their (b) primary and (c) co-receptors. The sequence alignments were generated using Clustal Omega Server[1] (http://www.ebi.ac.uk/Tools/msa/clustalo/). The β-bridge and β-strand assigned using DSSP[2] are shown for the top and bottom sequences. The secondary structures were from the IL-18/IL-18Rα/IL-18Rβ (3WO4), IL-1β/IL-1RI (4DEP) and IL-1RAcP (3O4O) structures.

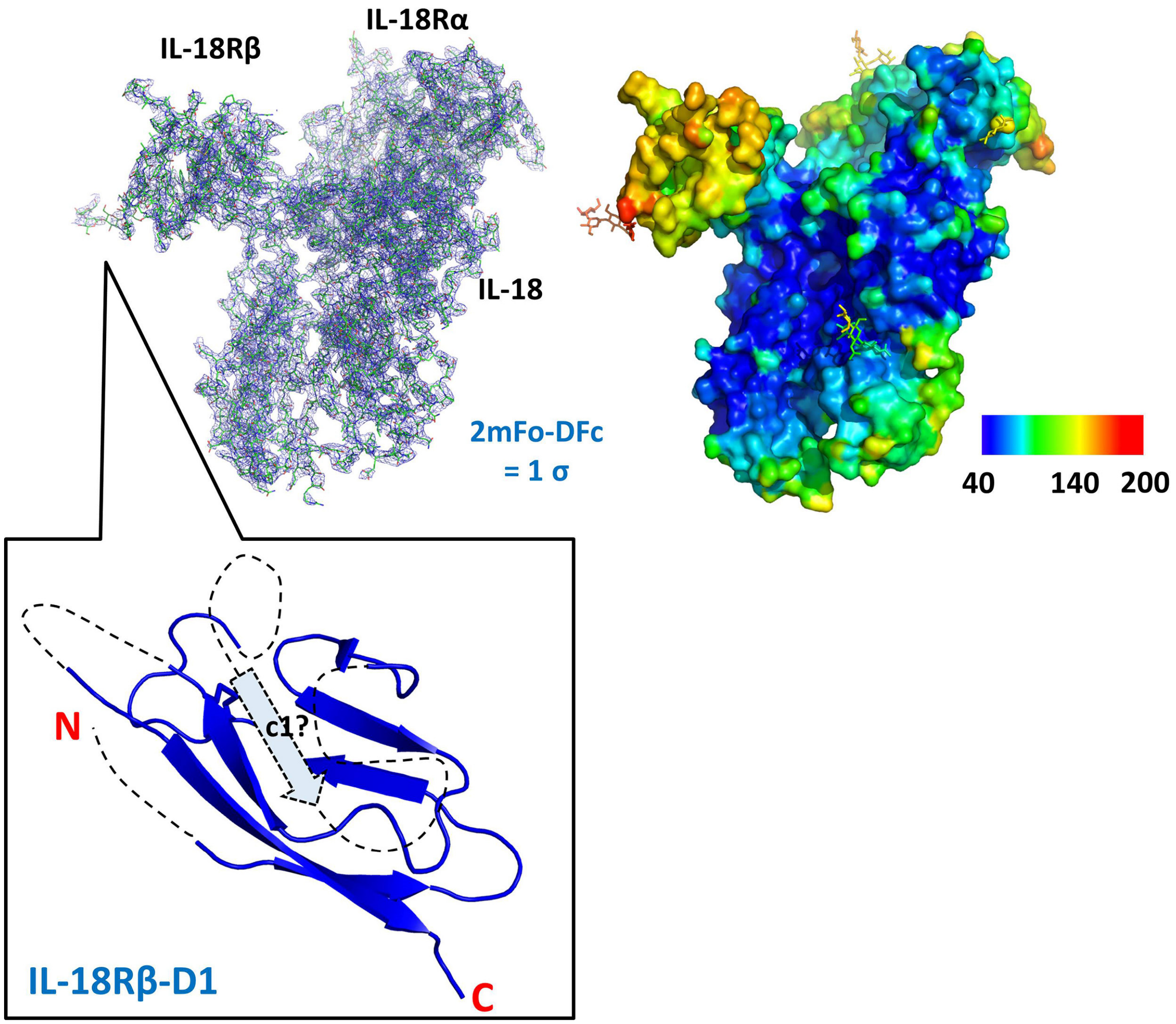

Supplementary Figure 2. Electron density map and temperature factor (B-factor) representation on the IL-18/IL-18Rα/IL-18Rβ structure.

The final 2mFo-DFc map contoured at 1.0 σ was overlain on the IL-18/IL-18Rα/IL-18Rβ structure, and the B-factors are colored on the structure surface with the minimum value 40 $Å^2$ (blue) and maximum value 200 $Å^2$ (red). The IL-18Rβ-D1 B-factor was clearly greater than for any other portion of the ternary complex. Based on the partly observed electron-density map as well as the similarity between IL-1RAcP and IL-18RAcP, the missing IL-18RAcP-D1 region is shown as a dotted line in a cartoon representation.

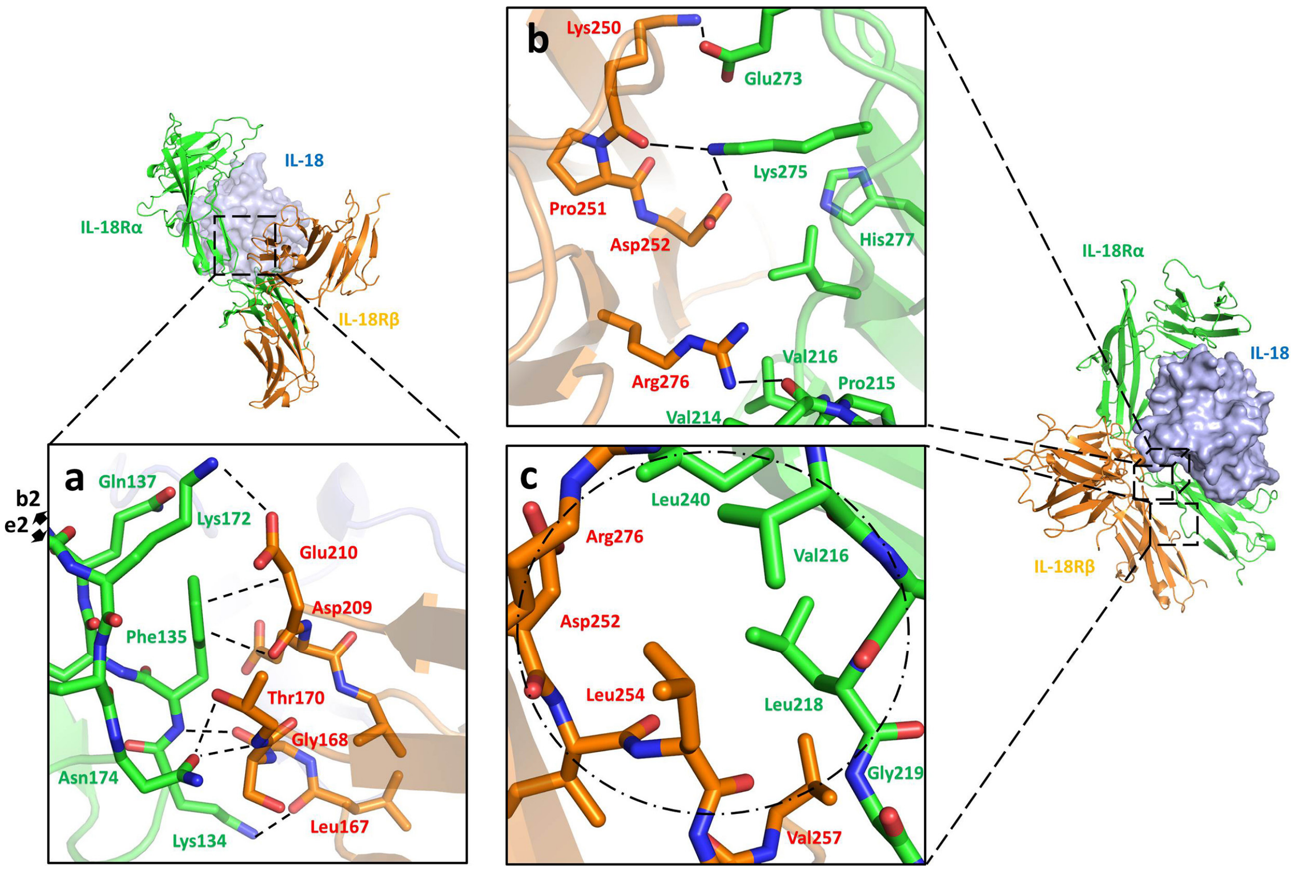

Supplementary Figure 3. The interface between IL-18Rα and IL-18Rβ in the signaling ternary complex.

(a) Structure of the D2:D2 contact region. The $Lys134^{R\alpha}$ side chain amino group forms hydrogen bonds with the $Leu167^{R\beta}$ backbone oxygen. $Phe135^{R\alpha}$ forms backbone-backbone hydrogen bonds with $Gly168^{R\beta}$ and a hydrophobic interaction with the $Asp209^{R\beta}$ and $Glu210^{R\beta}$ alkyl chains. $Lys172^{R\alpha}$ Nε-H forms close hydrogen bonds with $Asp209^{R\beta}$ Oδ. The $Asn174^{R\alpha}$ side chain amide forms multiple interactions with $Thr170^{R\beta}$ through the Oγ-H and backbone amide. The D3:D3 interaction is formed by (b) electrostatic or (c) aliphatic clusters. The electrostatic interaction is mainly composed of the $Glu273^{R\alpha}$, $Lys275^{R\alpha}$, $Lys250^{R\beta}$, $Asp252^{R\beta}$ and $Arg276^{R\beta}$ side chains, while the hydrophobic cluster is composed of $Val216^{R\alpha}$, $Leu218^{R\alpha}$, $Gly219^{R\alpha}$, $Leu254^{R\beta}$ and $Val257^{R\beta}$. The inter-receptor interfaces correspond to the IL-1β[3] system Site IV.

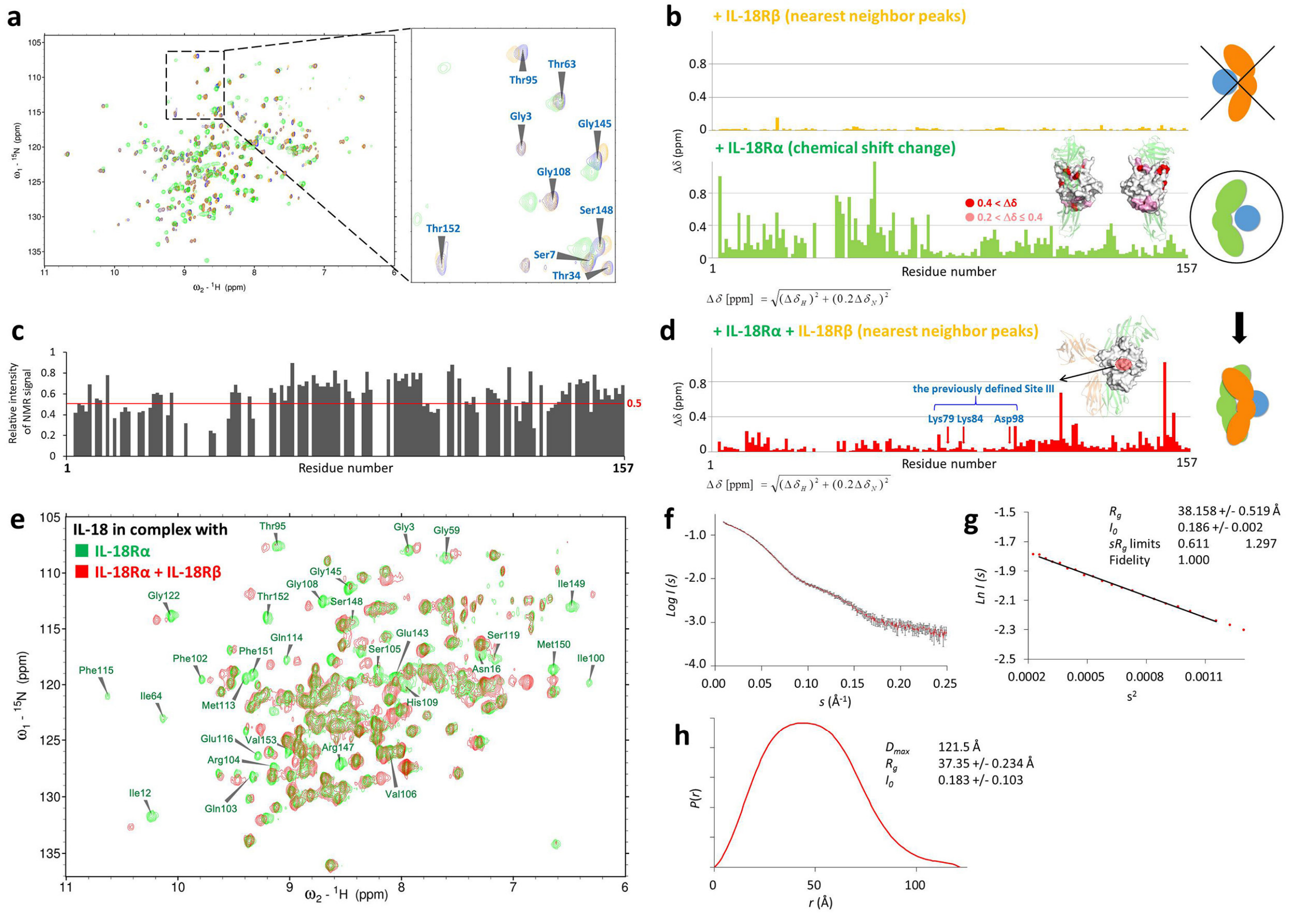

Supplementary Figure 4. Structural analysis of solution state IL-18/IL-18Rα/IL-18Rβ.
(a)-(e) Determining the receptor binding sites on IL-18 through solution NMR spectroscopy; (a) ^{1}H-^{15}N TROSY-HSQC spectra[4] for [^{2}H, ^{15}N]-IL18 with or without receptor titration. The spectra are from IL-18 (blue), IL-18+IL-18Rβ (orange) and IL-18+IL-18Rα (green), respectively. (b) IL-18 chemical shift perturbations upon adding (top) IL-18Rβ or (bottom) IL-18Rα. The amino acid residues perturbed at the bottom are colored on the IL-18/IL-18Rα crystal structure as indicated in the inset figures, which demonstrates that the solution binding mode is identical to the crystal structure binding mode. (c) Cross-saturation experiments[5] for [^{2}H, ^{15}N]-IL18/IL-18Rα. The intensity ratio (I_{2000ms}/I_{0ms}) plots for the assigned, separated cross peaks in the experiments. We assessed backbone the amide peaks for 108 out of 151 residues, except for the proline residues. (d) IL-18 chemical shift perturbation between IL-18/IL-18Rα to IL-18/IL-18Rα/IL-18Rβ. Significant changes were observed in the binding site for IL-18Rβ but not in the other region, including the previously defined Site III[6]. (e) The [^{2}H, ^{15}N]-IL-18/IL-18Rα spectral change upon binding IL-18Rβ. To clarify, we picked assigned peaks that clearly disappeared or moved from the binary to ternary complex. (f)-(h) IL-18/IL-18Rα/IL-18Rβ SAXS analysis; (f) the IL-18/IL-18Rα/IL-18Rβ scattering curve. S indicates the momentum transfer $4\pi\sin(\theta/2)/\lambda$; θ and λ are the scattering angle and X-ray wavelength, respectively. (g) Guinier approximation of the data in the low q region (qR_g<1.3). (h) Distance distribution function for the data. The D_{max} was determined through auto GNOM.

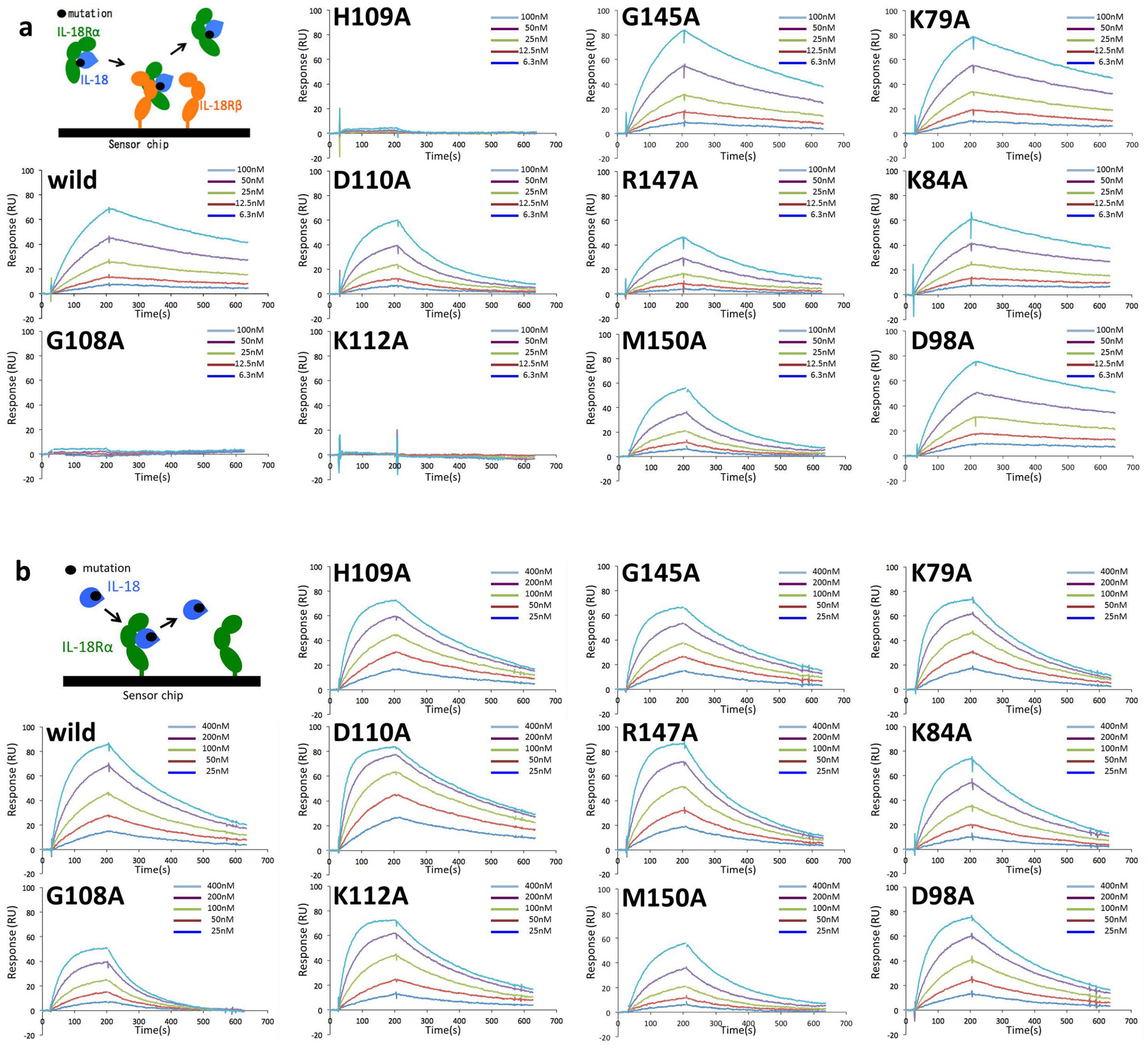

Supplementary Figure 5. SPR binding experiment with the IL-18 mutants.

(a) The sensorgrams show binding between the preformed IL-18 mutant/IL-18Rα complex and 6 × His tagged IL-18Rβ, which was immobilized on an NTA sensor chip. (b) Sensorgrams for IL-18 mutant binding to immobilized IL-18Rα. The sensorgrams are shown with a schematic figure for each experiment. The colored lines are the responses at 5 different concentrations.

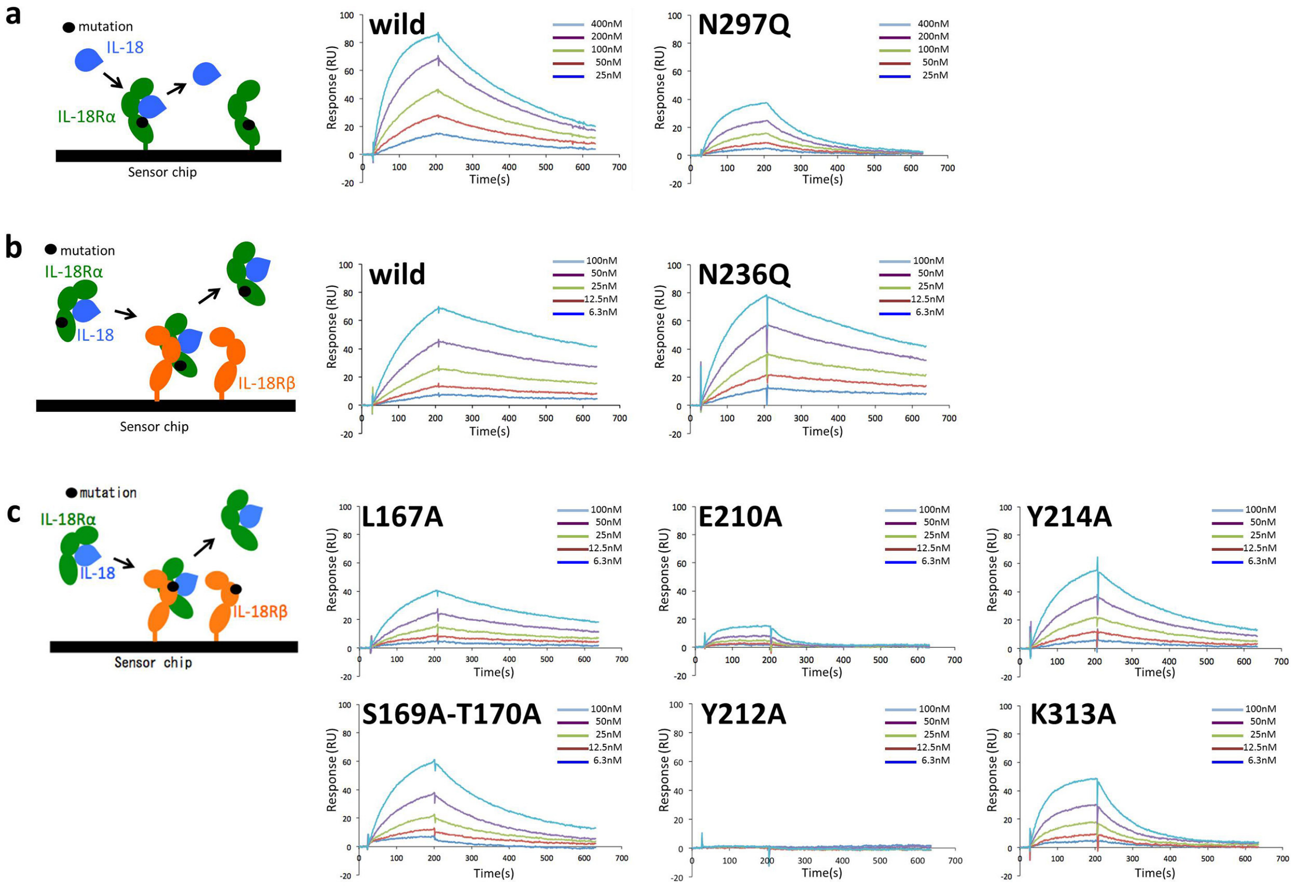

Supplementary Figure 6. SPR binding experiment with IL-18R mutants.

(a) IL-18 binding to the IL-18Rα-N297Q mutant. The absence of a sugar chain on N297$^{R\alpha}$ decreased the binding affinity by three-fold (Supplementary Table 1). (b) IL18/IL18Rα-N236Q binding to IL18Rβ. (c) The binding between the preformed binary IL-18/IL-18Rα complex and immobilized IL-18Rβ mutants. The sensorgrams are shown with a schematic figure for each experiment. The colored lines are the responses at 5 different concentrations.

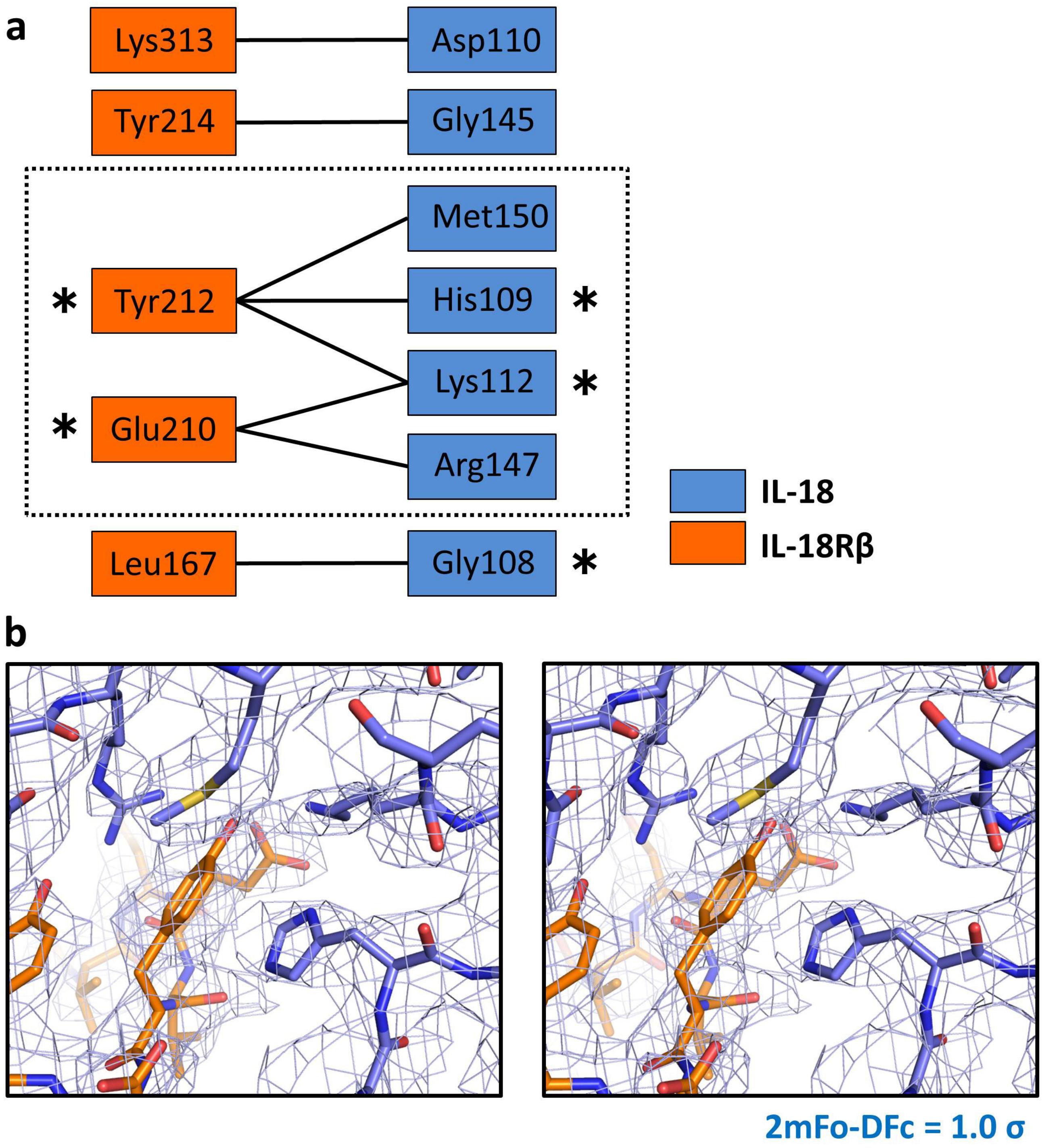

Supplementary Figure 7.

(a) The key residues at the IL-18 and IL-18Rβ interface, which were determined through the SPR experiment. Intermolecular structural interactions between amino acids side chains are represented by lines. The residues with apparent reduction in binding on SPR analysis are indicated by asterisks. The core of the interaction cluster was framed by a dashed box. (b) The stereo image (cross-eye) around the dashed box in Fig. 7a. The final 2mFo-DFc map contoured at 1.0 σ is overlain on the structure.

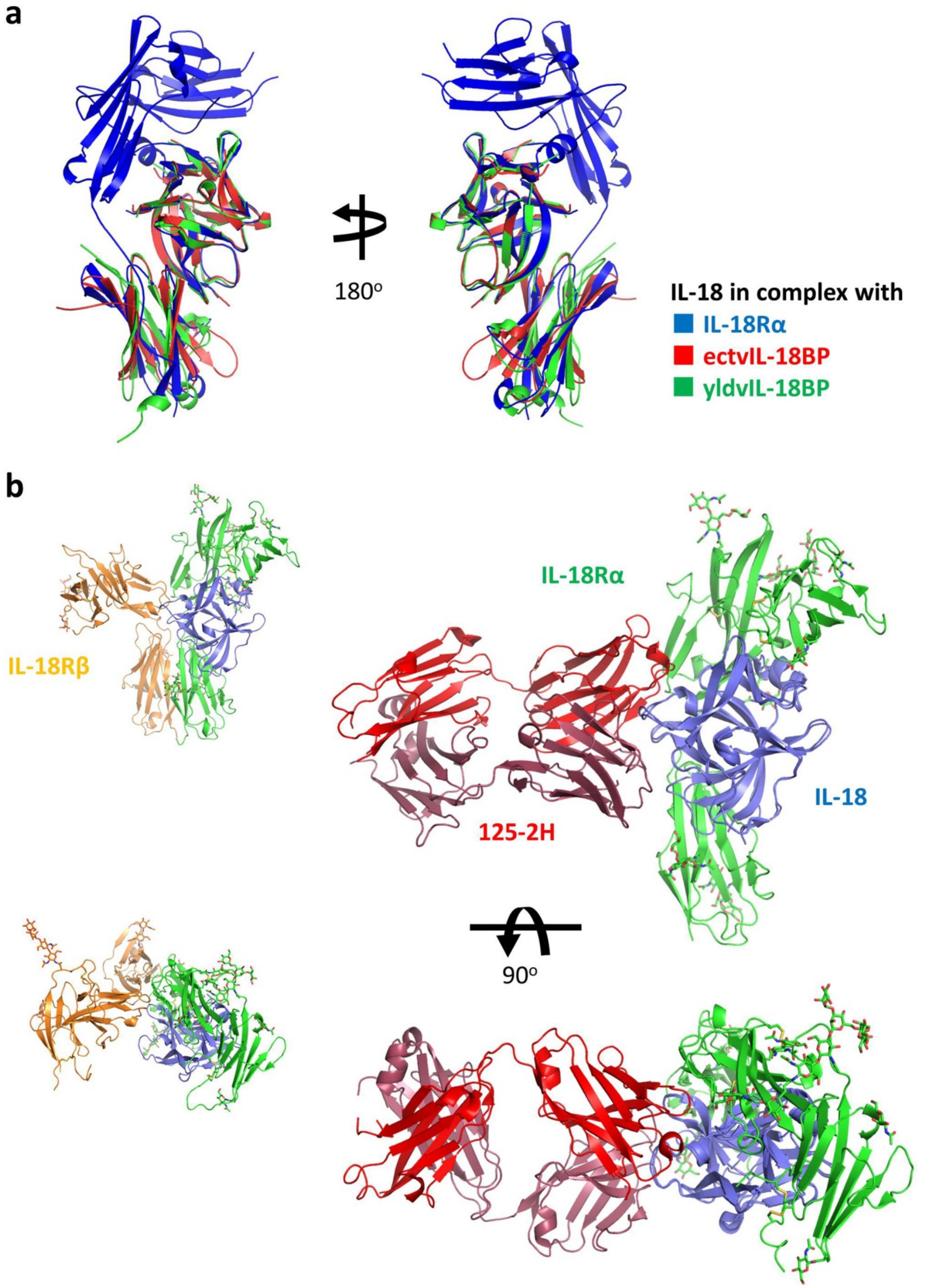

Supplementary Figure 8. IL-18-neutralization by the proteinous inhibitors.

(a) A comparison of the crystal structures for IL-18/IL-18Rα (blue) and IL-18 complexed with IL-18BPs (3F62:red and 4EEE:green). IL-18BP mimics IL-18Rα-D3 and binds the IL-18 binding Site II. (b) Ribbon representation of IL-18/125-2H superimposed with the binary complex IL-18/IL-18Rα. The anti-IL-18 antibody 125-2H binds to and covers IL-18 Site III, which inhibits IL-18Rβ binding.

Supplementary Table 1 Functional epitopes of the IL-18 and IL-18 receptors

Ligand: IL-18Rα WT
Analyte: **IL-18 mutant**

	K_{on} (10^4 M^{-1} s^{-1})	K_{off} (10^{-3} s)	K_d (10^{-8} M)
IL-18 WT(control)	4.8±0.1	3.3±0.1	6.9±0.2
IL-18 K79A	7.6±0.1	4.1±0.2	5.5±0.2
IL-18 K84A	3.9±0.1	3.7±0.1	9.4±0.2
IL-18 D98A	5.4±0.2	3.4±0.1	6.4±0.2
IL-18 G108A	5.2±0.3	9.5±0.2	18.4±1.4
IL-18 H109A	6.2±0.4	3.0±0.2	4.8±0.1
IL-18 D110A	10.6±0.1	2.4±0.1	2.3±0.1
IL-18 K112A	6.4±1.1	3.3±0.3	5.1±0.5
IL-18 G145A	5.9±0.6	5.9±0.6	5.7±0.5
IL-18 R147A	6.8±0.1	6.8±0.1	6.7±0.4
IL-18 M150A	5.7±0.2	5.7±0.2	5.9±0.4

Ligand: **IL-18Rα mutant**
Analyte: IL-18 WT

	K_{on} (10^4 M^{-1} s^{-1})	K_{off} (10^{-3} s)	K_d (10^{-8} M)
IL-18Rα WT(control)	4.8±0.1	3.3±0.1	6.9±0.2
IL-18Rα N297Q	3.4±0.4	8.9±0.5	25.8±2.6
IL-18Rα T23A-R25A-H27A-T29A		No binding	
IL-18Rα R123A-T126A-S127A-K128A		No binding	
IL-18Rα E253A-E254A-E263A		No binding	

Ligand: IL-18Rβ WT
Analyte: **IL-18 mutant**/IL-18Rα WT complex

	K_{on} (10^4 M^{-1} s^{-1})	K_{off} (10^{-3} s)	K_d (10^{-8} M)
IL-18 WT(control)	1.1±0.1	1.6±0.1	1.5±0.1
IL-18 K79A	1.3±0.1	1.7±0.2	1.3±0.0
IL-18 K84A	1.0±0.1	1.8±0.3	1.8±0.1
IL-18 D98A	1.2±0.1	1.6±0.2	1.3±0.1
IL-18 G108A		No binding	
IL-18 H109A		No binding	
IL-18 D110A	1.4±0.1	5.6±0.8	5.6±0.8
IL-18 K112A		No binding	
IL-18 G145A	8.2±0.3	2.8±0.1	2.8±0.1
IL-18 R147A	6.9±0.4	5.7±0.3	5.7±0.3
IL-18 M150A	8.9±0.9	6.0±0.5	6.0±0.5

Ligand: IL-18Rβ WT
Analyte: IL-18WT/**IL-18Rα mutant** complex

	K_{on} (10^4 M^{-1} s^{-1})	K_{off} (10^{-3} s)	K_d (10^{-8} M)
IL-18Rα WT(control)	1.1±0.1	1.6±0.1	1.5±0.1
IL-18Rα N236Q	1.2±0.1	2.0±0.3	1.7±0.3

Ligand: **IL-18Rβ mutant**
Analyte: IL-18 WT/IL-18Rα WT complex

	K_{on} (10^4 M^{-1} s^{-1})	K_{off} (10^{-3} s)	K_d (10^{-8} M)
IL-18Rβ WT(control)	1.1±0.1	1.6±0.1	1.5±0.1
IL-18Rβ L167A	7.6±1.2	2.5±0.1	3.3±0.4
IL-18RβS169A-T170A	6.8±0.6	5.2±0.2	7.7±0.7
IL-18Rβ E210A	7.1±1.8	22.2±0.9	32.7±0.9
IL-18Rβ Y212A		No binding	
IL-18Rβ Y214A	9.7±0.9	4.8±0.3	5.0±0.5
IL-18Rβ K313A	10.3±1.0	11.2±0.8	10.9 ±0.9

Ligand: The interactant collected on the sensor surface
Analyte: The interactant in free solution
WT: wild type

The values are the means ± standard deviations of the results from 3 independent experiments

SUPPLEMENTARY REFERENCES

1. Sievers, F. *et al.* Fast, scalable generation of high-quality protein multiple sequence alignments using Clustal Omega. *Mol. Syst. Biol.* **7,** 539 (2011).

2. Joosten, R. P. *et al.* A series of PDB related databases for everyday needs. *Nucleic Acids Res.* **39,** D411–9 (2011).

3. Wang, D. *et al.* Structural insights into the assembly and activation of IL-1β with its receptors. *Nat. Immunol.* **11,** 905–11 (2010).

4. Pervushin, K., Riek, R., Wider, G. & Wüthrich, K. Attenuated T2 relaxation by mutual cancellation of dipole-dipole coupling and chemical shift anisotropy indicates an avenue to NMR structures of very large biological macromolecules in solution. *Proc. Natl. Acad. Sci. U. S. A.* **94,** 12366–71 (1997).

5. Takahashi, H., Nakanishi, T., Kami, K., Arata, Y. & Shimada, I. A novel NMR method for determining the interfaces of large protein-protein complexes. *Nat. Struct. Biol.* **7,** 220–3 (2000).

6. Kato, Z. *et al.* The structure and binding mode of interleukin-18. *Nat. Struct. Biol.* **10,** 966–71 (2003).
